# Supplementary material for: Deep multiple instance learning versus conventional deep single instance learning for interpretable oral cancer detection
Source: PLoS One. 2024 Apr 30;19(4):e0302169. doi: 10.1371/journal.pone.0302169 (PMC11060593; doi:10.1371/journal.pone.0302169)
Supplement: S1 Dataset — (PDF) [file pone.0302169.s001.pdf]

The PAP-QMNIST datasets generated and analyzed during the current study are available in the Zenodo repository, doi 10.5281/zenodo.7020311, together with the code for generating PAP-QMNIST data.
