# Supplementary material for: Deep multiple instance learning versus conventional deep single instance learning for interpretable oral cancer detection
Source: PLoS One. 2024 Apr 30;19(4):e0302169. doi: 10.1371/journal.pone.0302169 (PMC11060593; doi:10.1371/journal.pone.0302169)
Supplement: S1 Table — (PDF) [file pone.0302169.s002.pdf]

Transformation [18]	Parameters
Resize	height=80, width=80, probability=1
Rotate	angle limit=(-90,90), probability=1
GaussNoise	variance range for noise (2,5), probability=0.1
HorizontalFlip	probability=0.5
Blur	blur limit=5, probability=0.15
RandomBrightnessContrast	factor range (-0.2,0.2), probability=0.8
MotionBlur	blur limit range (3,3), probability=0.05
RandomFog	lower coefficient=0.3, upper coefficient=0.4, alpha coefficient=0.08, probability=0.05
