# Supplementary material for: Deep multiple instance learning versus conventional deep single instance learning for interpretable oral cancer detection
Source: PLoS One. 2024 Apr 30;19(4):e0302169. doi: 10.1371/journal.pone.0302169 (PMC11060593; doi:10.1371/journal.pone.0302169)
Supplement: S2 Table — (PDF) [file pone.0302169.s003.pdf]

	Description of layer	Purpose of layer
1	Conv2d(3, 20, kernel size=(5, 5), stride=(1, 1))	feature extraction
2	ReLU	feature extraction
3	MaxPool2d(kernel size=2, stride=2, padding=0, dilation=1)	feature extraction
4	Conv2d(20, 50, kernel size=(5, 5), stride=(1, 1))	feature extraction
5	ReLU	feature extraction
6	MaxPool2d(kernel size=2, stride=2, padding=0, dilation=1)	feature extraction
7	Linear(input features=$50 \times 17 \times 17$, output features=500, bias=True)	feature extraction
8	ReLU	feature extraction
9	Linear(input features=500, output features=128, bias=True)	attention mechanism
10	Tanh	attention mechanism
11	Linear(input features=128, output features=1, bias=True)	attention mechanism
12	Linear(input features=500, output features=1, bias=True)	classification
13	Sigmoid	classification
