# Supplementary material for: Deep multiple instance learning versus conventional deep single instance learning for interpretable oral cancer detection
Source: PLoS One. 2024 Apr 30;19(4):e0302169. doi: 10.1371/journal.pone.0302169 (PMC11060593; doi:10.1371/journal.pone.0302169)
Supplement: S4 Table — (PDF) [file pone.0302169.s005.pdf]

	Learning rate		Weight decay		Optimizer	(β_1, β_2)
	ABMIL	SIL	ABMIL	SIL		
LeNet	$5 \cdot 10^{-5}$	10^{-4}	10^{-6}	10^{-4}	Adam	(0.9,0.999)
ResNet18	$5 \cdot 10^{-6}$	10^{-4}	10^{-5}	10^{-6}		
SqueezeNet	$5 \cdot 10^{-5}$	10^{-4}	10^{-5}	10^{-6}		
