# Supplementary material for: Deep multiple instance learning versus conventional deep single instance learning for interpretable oral cancer detection
Source: PLoS One. 2024 Apr 30;19(4):e0302169. doi: 10.1371/journal.pone.0302169 (PMC11060593; doi:10.1371/journal.pone.0302169)
Supplement: S1 Appendix — (PDF) [file pone.0302169.s006.pdf]

Architecture for LeNet-based ABMIL with sampling is taken from [9] and shown in S2 Table. Architecture for LeNet-based SIL is a combination of the feature extraction part of LeNet-based ABMIL with sampling (layers 1-8 in S2 Table) and layers for binary classification (layers 9-10) (see S3 Table). Architecture for ResNet18-based SIL is as ResNet18 model in [19]. Architecture for SqueezeNet-based SIL is the PyTorch available model with version 1.1 from the official SqueezeNet repo `https://github.com/forresti/SqueezeNet/tree/master/SqueezeNet_v1.1`. Architectures for ResNet18-based and SqueezeNet-based ABMIL with sampling are composed in the same way as LeNet-based ABMIL with sampling model, i.e., as a sequence of feature extraction part, attention mechanism, and classifier. Architectures for ResNet18-based and SqueezeNet-based ABMIL with sampling include (i) the feature extraction part (no classification layers) of original ResNet18 [19], and SqueezeNet version 1.1 mentioned above, respectively, for feature extraction, (ii) the MIL attention layers as in S2 Table (with the number of nodes in the attention layer equal to 524 and 128, correspondingly) for obtaining attention weights, and (iii) classification layers for classifying bags (with the input feature size of 2048 and 512, correspondingly).
