# Supplementary material for: Deep multiple instance learning versus conventional deep single instance learning for interpretable oral cancer detection
Source: PLoS One. 2024 Apr 30;19(4):e0302169. doi: 10.1371/journal.pone.0302169 (PMC11060593; doi:10.1371/journal.pone.0302169)
Supplement: S2 Appendix — (PDF) [file pone.0302169.s007.pdf]

To ensure an appropriate choice of mini-batch size, we have evaluated SIL performance for mini-batch sizes of 56, 500, 1200, and 2500 images, allowing the model to be trained for 1500 epochs instead of 150 epochs and with the same stopping criteria. The increased number of epochs for training was chosen due to the observation that models trained with larger mini-batch sizes were converging slower than models trained with a mini-batch size of 56. We observe (see S1 Fig) that the performance does not differ a lot depending on the mini-batch size, and there is no visible trend. Interestingly, for the models trained with mini-batches of 56 images, there are several convergence plateaus for the validation set where one may select to pick a model for inference, however, the earlier solution (found within the first 150 epochs) generalizes better on the test sets (early stopping). Considering the additional required computational resources for training with larger mini-batch sizes, along with a better performance from early stopping, we conclude that mini-batch size of 56 images and the overall number of training epochs equal to 150 for SIL are most suitable.
