# Supplementary figures and images for: Deep multiple instance learning versus conventional deep single instance learning for interpretable oral cancer detection
Source: PLoS One. 2024 Apr 30;19(4):e0302169. doi: 10.1371/journal.pone.0302169 (PMC11060593; doi:10.1371/journal.pone.0302169)

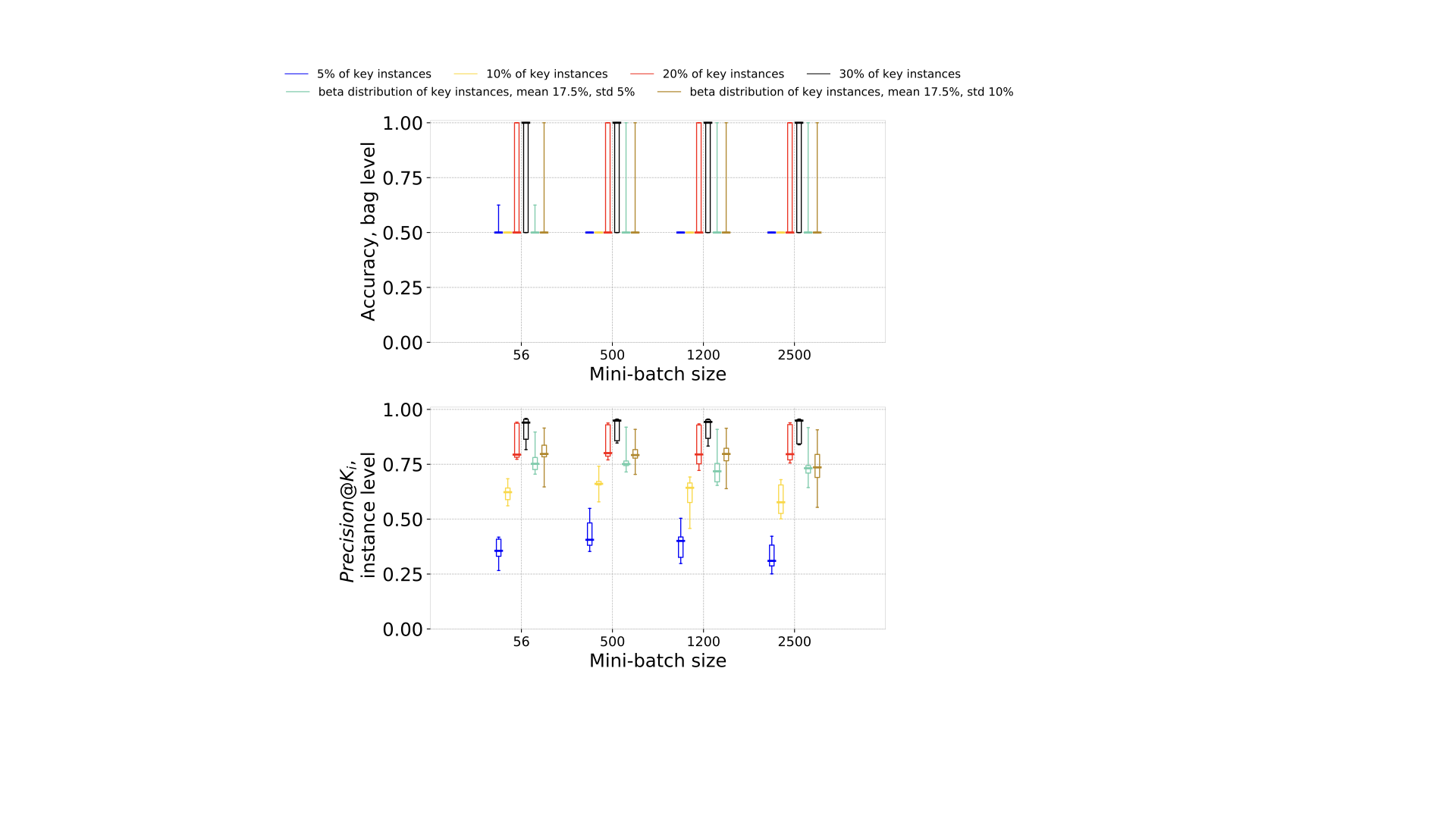

Supplement: S1 Fig — Four mini-batch sizes are indicated on the x-axis. The box plots display minimum, first quartile, median, third quartile, and maximum (the five-number summary) over 9 folds. (TIF) [file pone.0302169.s008.tif]
