# Supplementary material for: Deep multiple instance learning versus conventional deep single instance learning for interpretable oral cancer detection
Source: PLoS One. 2024 Apr 30;19(4):e0302169. doi: 10.1371/journal.pone.0302169 (PMC11060593; doi:10.1371/journal.pone.0302169)
Supplement: Attachment — Submitted filename: PONE-D-23-20689.pdf [file pone.0302169.s009.pdf]

PLOS ONE

Deep multiple instance learning versus conventional deep single instance learning for interpretable oral cancer detection

--Manuscript Draft--

**Manuscript Number:**	PONE-D-23-20689
**Article Type:**	Research Article
**Full Title:**	Deep multiple instance learning versus conventional deep single instance learning for interpretable oral cancer detection
**Short Title:**	Multiple instance learning versus conventional single instance learning for oral cancer detection
**Corresponding Author:**	Nadezhda Koriakina<br>Uppsala Universitet<br>Uppsala, SWEDEN
**Keywords:**	Multiple instance learning, single instance learning, interpretability, oral cancer
**Abstract:**	The current medical standard for setting an oral cancer (OC) diagnosis is histological examination of a tissue sample taken from the oral cavity. This process is time-consuming and more invasive than an alternative approach of acquiring a brush sample followed by cytological analysis. Using a microscope, skilled cytotechnologists are able to detect changes due to malignancy; however, introducing this approach into clinical routine is associated with challenges such as a lack of resources and experts.<br>To design a trustworthy OC detection system that can assist cytotechnologists, we are interested in AI-based methods that can reliably detect cancer, given only per-patient labels (thereby minimizing annotation bias), and also provide information regarding which cells are most relevant for the diagnosis (thereby enabling supervision and understanding).<br>In this study, we perform a comparison of two approaches suitable for OC detection and interpretation: (i) conventional single instance learning (SIL) approach and (ii) a modern multiple instance learning (MIL) method.<br>To facilitate systematic evaluation of the considered approaches, we, in addition to a real OC dataset with patient-level ground truth annotations, also introduce a synthetic dataset -- PAP-QMNIST. This dataset serves as a proxy model of a real OC dataset, while offering access to reliable per-instance ground truth.<br>For both OC and PAP-QMNIST data, we evaluate performance of the methods utilizing three different neural network architectures.<br>Our study indicates, somewhat surprisingly, that on PAP-QMNIST, the SIL approach performs better, on average, than the MIL approach. Performance at the bag level on real cytological OC data is similar for both methods, yet the SIL approach performs better on average. Visual examination by cytotechnologist indicates that the methods manage to identify cells which deviate from normality, including malignant cells as well as those suspicious for dysplasia.<br>We share the code as open source at https://github.com/MIDA-group/OralCancerMILvsSIL
**Order of Authors:**	Nadezhda Koriakina
	Joakim Lindblad
	Natasa Sladoje
	Vladimir Basic
**Additional Information:**	
**Question**	**Response**
**Financial Disclosure**<br><br>Enter a financial disclosure statement that describes the sources of funding for the	This work is supported by: Sweden’s Innovation Agency (VINNOVA) https://www.vinnova.se/en/apply-for-funding/funded-projects/, grants 2017-02447 (J.L.) and 2020-03611 (J.L.), and the Swedish Research Council https://www.vr.se/english/swecris.html#/, grant 2017-04385(J.L.). A part of the

Powered by Editorial Manager® and ProduXion Manager® from Aries Systems Corporation

work included in this submission. Review the submission guidelines for detailed requirements. View published research articles from *PLOS ONE* for specific examples.<br><br>This statement is required for submission and **will appear in the published article** if the submission is accepted. Please make sure it is accurate.<br><br>**Unfunded studies**<br>Enter: *The author(s) received no specific funding for this work.*<br><br>**Funded studies**<br>Enter a statement with the following details:<br>• Initials of the authors who received each award<br>• Grant numbers awarded to each author<br>• The full name of each funder<br>• URL of each funder website<br>• Did the sponsors or funders play any role in the study design, data collection and analysis, decision to publish, or preparation of the manuscript?<br>• **NO** - Include this sentence at the end of your statement: *The funders had no role in study design, data collection and analysis, decision to publish, or preparation of the manuscript.*<br>• **YES** - Specify the role(s) played.<br><br>* typeset	computations was enabled by resources provided by the National Academic Infrastructure for Supercomputing in Sweden (NAISS) and the Swedish National Infrastructure for Computing (SNIC) at Chalmers Centre for Computational Science and Engineering (C3SE), partially funded by the Swedish Research Council through grant agreements no. 2022-06725 and no. 2018-05973. The funders had no role in study design, data collection and analysis, decision to publish, or preparation of the manuscript.
**Competing Interests**<br><br>Use the instructions below to enter a competing interest statement for this submission. On behalf of all authors, disclose any competing interests that could be perceived to bias this work—acknowledging all financial support and any other relevant financial or non-financial competing interests.<br><br>This statement is required for submission and **will appear in the published article** if the submission is accepted. Please make sure it is accurate and that any funding sources listed in your Funding Information	The authors have declared that no competing interests exist.

later in the submission form are also declared in your Financial Disclosure statement.<br><br>View published research articles from *PLOS ONE* for specific examples.<br><br>**NO authors have competing interests**<br><br>Enter: *The authors have declared that no competing interests exist.*<br><br>**Authors with competing interests**<br><br>Enter competing interest details beginning with this statement:<br><br>*I have read the journal's policy and the authors of this manuscript have the following competing interests: [insert competing interests here]*<br><br>* typeset	
**Ethics Statement**<br><br>Enter an ethics statement for this submission. This statement is required if the study involved:<br><br>• Human participants<br>• Human specimens or tissue<br>• Vertebrate animals or cephalopods<br>• Vertebrate embryos or tissues<br>• Field research<br><br>Write "N/A" if the submission does not require an ethics statement.<br><br>General guidance is provided below. Consult the submission guidelines for detailed instructions. **Make sure that all information entered here is included in the Methods section of the manuscript.**	The study was performed in compliance with the Declaration of Helsinki and approved by the Ethical Review Board Stockholm Sweden (2015-1213-31 and 2019-00349). Informed written consent was obtained from all participants. The study does not involve minors. Data were accessed for research purposes during 2020 and 2021. Researcher carrying out the project, Christina Runow Stark, has changed the job since then therefore is not an author of the paper. The analysis is not based on third party data.

**Format for specific study types**<br><br>**Human Subject Research (involving human participants and/or tissue)**<br>• Give the name of the institutional review board or ethics committee that approved the study<br>• Include the approval number and/or a statement indicating approval of this research<br>• Indicate the form of consent obtained (written/oral) or the reason that consent was not obtained (e.g. the data were analyzed anonymously)<br><br>**Animal Research (involving vertebrate animals, embryos or tissues)**<br>• Provide the name of the Institutional Animal Care and Use Committee (IACUC) or other relevant ethics board that reviewed the study protocol, and indicate whether they approved this research or granted a formal waiver of ethical approval<br>• Include an approval number if one was obtained<br>• If the study involved *non-human primates*, add *additional details* about animal welfare and steps taken to ameliorate suffering<br>• If anesthesia, euthanasia, or any kind of animal sacrifice is part of the study, include briefly which substances and/or methods were applied<br><br>**Field Research**<br><br>Include the following details if this study involves the collection of plant, animal, or other materials from a natural setting:<br>• Field permit number<br>• Name of the institution or relevant body that granted permission	
**Data Availability**<br><br>Authors are required to make all data underlying the findings described fully available, without restriction, and from the time of publication. PLOS allows rare exceptions to address legal and ethical concerns. See the PLOS Data Policy and FAQ for detailed information.	No - some restrictions will apply

A Data Availability Statement describing where the data can be found is required at submission. Your answers to this question constitute the Data Availability Statement and **will be published in the article**, if accepted.<br><br>**Important:** Stating 'data available on request from the author' is not sufficient. If your data are only available upon request, select 'No' for the first question and explain your exceptional situation in the text box.<br><br>Do the authors confirm that all data underlying the findings described in their manuscript are fully available without restriction?	
**Describe where the data may be found in full sentences. If you are copying our sample text, replace any instances of XXX with the appropriate details.**<br><br>• If the data are **held or will be held in a public repository**, include URLs, accession numbers or DOIs. If this information will only be available after acceptance, indicate this by ticking the box below. For example: *All XXX files are available from the XXX database (accession number(s) XXX, XXX.).*<br>• If the data are all contained **within the manuscript and/or Supporting Information files**, enter the following: *All relevant data are within the manuscript and its Supporting Information files.*<br>• If neither of these applies but you are able to provide **details of access elsewhere**, with or without limitations, please do so. For example:<br><br>*Data cannot be shared publicly because of [XXX]. Data are available from the XXX Institutional Data Access / Ethics Committee (contact via XXX) for researchers who meet the criteria for access to confidential data.*<br><br>*The data underlying the results presented in the study are available from (include the name of the third party*	PAP-QMNIST data: https://doi.org/10.5281/zenodo.7020312; codes for framework: https://github.com/MIDA-group/OralCancerMILvsSIL

and contact information or URL).<br>• This text is appropriate if the data are owned by a third party and authors do not have permission to share the data.<br><br>* typeset	
Additional data availability information:	Tick here if the URLs/accession numbers/DOIs will be available only after acceptance of the manuscript for publication so that we can ensure their inclusion before publication.

Powered by Editorial Manager® and ProduXion Manager® from Aries Systems Corporation

Manuscript

Click here to access/download;Manuscript;SciRep_to_PLOS_possible_resub

Deep multiple instance learning versus conventional deep single instance learning for interpretable oral cancer detection

Nadezhda Koriakina[1*], Nataša Sladoje[1], Vladimir Bašić[2,3], Joakim Lindblad[1],

1 Centre for Image Analysis, Dept. of Information Technology, Uppsala University, Uppsala, Sweden
2 Dept. of Natural Science and Biomedicine, School of Health and Welfare, Jönköping University, Jönköping, Sweden
3 Clinical Research Center Dalarna, Uppsala University, Falun, Sweden

¤Current Address: Centre for Image Analysis, Dept. of Information Technology, Uppsala University, Uppsala, Sweden
* nadezhda.koriakina@it.uu.se

Abstract

The current medical standard for setting an oral cancer (OC) diagnosis is histological examination of a tissue sample taken from the oral cavity. This process is time-consuming and more invasive than an alternative approach of acquiring a brush sample followed by cytological analysis. Using a microscope, skilled cytotechnologists are able to detect changes due to malignancy; however, introducing this approach into clinical routine is associated with challenges such as a lack of resources and experts. To design a trustworthy OC detection system that can assist cytotechnologists, we are interested in AI-based methods that can reliably detect cancer, given only per-patient labels (thereby minimizing annotation bias), and also provide information regarding which cells are most relevant for the diagnosis (thereby enabling supervision and understanding). In this study, we perform a comparison of two approaches suitable for OC detection and interpretation: (i) conventional single instance learning (SIL) approach and (ii) a modern multiple instance learning (MIL) method. To facilitate systematic evaluation of the considered approaches, we, in addition to a real OC dataset with patient-level ground truth annotations, also introduce a synthetic dataset – PAP-QMNIST. This dataset serves as a proxy model of a real OC dataset, while offering access to reliable per-instance ground truth. For both OC and PAP-QMNIST data, we evaluate performance of the methods utilizing three different neural network architectures. Our study indicates, somewhat surprisingly, that on PAP-QMNIST, the SIL approach performs better, on average, than the MIL approach. Performance at the bag level on real cytological OC data is similar for both methods, yet the SIL approach performs better on average. Visual examination by cytotechnologist indicates that the methods manage to identify cells which deviate from normality, including malignant cells as well as those suspicious for dysplasia. We share the code as open source at `https://github.com/MIDA-group/OralCancerMILvsSIL`

Author summary

J.L., N.S. and N.K. conceived the experiments, N.K. conducted the experiments. V.B. performed a per-cell evaluation on cytological data. All authors reviewed the manuscript.

Introduction

Cancers of the oral cavity and oropharynx are on the list of the most common malignancies in the world. Early detection of cancer is highly desirable as it is a premise of successful treatment. Current gold standard for confirming a diagnosis is histological examination of a tissue biopsy sample, which is time consuming and painful for the patient. An alternative is to develop painless methods which rely on taking brush samples from the oral cavity of the patients and subsequently analyse the collected and suitably prepared cytological data. A trained cytologist is able to detect abnormalities in samples acquired from patients with malignancy by carefully examining the cells by a microscope. However, this is a very difficult task; a recent study indicates sensitivity and specificity reached by human experts *on the patient-level diagnosis* being not more than 80% and 86%, respectively, in oral cancer cytology screening [1]. Steps towards improving diagnostic accuracy, but also steps towards improved understanding of the malignancy, its causes and consequences, are therefore very valuable.

Automated analysis of cytological data can increase efficiency of the process to a level that opens possibilities for population-wide screening, towards early cancer detection. Deep learning (DL) based image classification methods have shown ability to detect differences between malignant versus healthy samples, without the need for very difficult and time consuming labeling of each individual cell [2]. Systems based on such methods for oral cancer (OC) detection could assist cytologists, given that the method is reliable enough. For successful adoption of DL-supported cancer detection in healthcare, it is not sufficient for a classifier to provide just an answer 'yes' or 'no'. In order to trust the classifier's predictions, a human expert needs information about *why* the system reaches a certain decision [3]. An example of such explanatory information is a provided set of cells identified by the system as malignant, leading to the patient-level decision of detected cancer. However, (even) to evaluate such information becomes a challenging task when only per-patient, and not per-cell, labels are available, as is typically the case for cytology OC data. While the relatively few, subjectively judged, per-cell annotations provided by a cytotechnologist are not sufficient for training, thorough evaluation, nor reliable interpretation of the DL classification methods, they are still useful for observing similarities and differences between human and machine findings.

Aiming towards efficient and trustworthy cytology-based OC detection, we are interested in DL based methods that are able to learn from only patient-level labels and demonstrate good patient-level performance, while also providing information about the importance of individual cells for a diagnosis, thereby facilitating human interpretation and supervision of the decision made by the DL method.

To tackle this challenge, we are following emerging approaches taken in Whole Slide Image (WSI) analysis for (bio)medical applications [4], where labeling of all parts of a WSI is not feasible and only weak labels are available. More precisely, we are addressing OC detection as a weakly supervised learning problem. Due to the large size of WSIs, the solutions typically involve dividing each WSI into many smaller patches, which are further processed. We utilize this idea too, while still striving to preserve and use holistic, slide-level information. Publicly available datasets for computational cytology do not meet the criteria of preserving slide-level information and are composed of preselected cells or tiles. Other (non-public) cytology datasets that are comprised of WSIs are most often suitable for detection of cervical, not oral cancer [5]. For our collected OC dataset, we choose an automated approach which allows to consider all the cells in the WSIs. This is in line with the clinical routine, where cytotechnologists typically look at a whole WSI to find a few abnormal cells.

Multiple instance learning (MIL) is a type of weakly supervised learning which can offer methods suitable for OC detection [6]. While *bags* are composed of (multiple) instances, the core idea of MIL is to treat the bags as individual objects, each with a

well-defined label. When MIL is used for binary classification, the aim is to classify negative bags against positive bags; a bag is classified as positive if it contains at least one positive (key) instance, whereas a negative bag does not comprise any key instances.

WSI analysis with patient-level labels can be seen as an example of MIL, with a set of labeled bags (patient samples), such that a bag is labeled positive if at least one instance (one cell) in it is positive (malignant). An alternative to MIL is a conventional deep single instance learning (SIL) approach. In the absence of per-cell annotations, each cell is given a label corresponding to the label of the sample/patient it comes from. Since a sample acquired from a patient with a malignancy may include very many normal (non-malignant) cells, to use a patient-level label as an indicator of malignancy on the cell-level constitutes a very unreliable label; this approach can therefore be seen as another, differently formulated, weakly supervised learning task.

In this study, we compare two methods, one MIL-based and one conventional SIL-based approach. The methods are conceptually different, but are both based on deep convolutional neural networks (CNN). Our main interest is to: (i) compare the performance of the two weakly supervised end-to-end methods, on the per-patient level, as well as on the per-cell level prediction; and (ii) explore the decision-making strategies of both approaches by evaluating the networks' ability to identify the key instances, *i.e.*, the diagnostically most relevant cells.

To be able to reliably evaluate the observed methods, we have created PAP-QMNIST, a synthetic dataset that mimics several properties of WSI OC data relevant for training a CNN in both MIL and SIL regimes, such as cell image size, color distribution, arbitrary rotation of cells, amount of blur and noise, large and varied number of instances per bag. We note that creating a (more realistic) synthetic dataset composed of images mimicking cells with reliable ground truth (GT) annotation at the instance (cell) level is not feasible because properties that distinguish malignant versus healthy cells are not fully known. A main advantage of PAP-QMNIST is that it offers access to reliable ground truth annotation at the instance (cell) level in combination with being visually interpretable for non-experts. The PAP-QMNIST dataset allows us to analyze the behavior of methods, e.g. when the methods start to fail or demonstrate dissimilar results. Such analysis is much more challenging on real data with limited GT and complex interpretation. We envision that the PAP-QMNIST dataset may be useful for similar studies by other researchers in the field.

The main contributions of our work are: (i) We introduce a new dataset, PAP-QMNIST, as a proxy model with similar distribution as the cytological data, with a well defined and visually assessable ground truth; (ii) We use PAP-QMNIST to evaluate the performance of two different approaches for weakly supervised malignancy detection; (iii) We compare the two observed approaches on real medical cytological OC WSI data, focusing on both per-patient and per-cell performance, and analyze to what extent the findings on the synthetic data transfer to the real cytology data; (iv) We observe that it is possible to detect malignancy (key instances) in both synthetic and real data using both observed approaches, and we observe similar performance at patient (bag) level classification for both approaches. We also observe that SIL approach outperforms MIL approach in the detection of malignant cells (key instances); (v) By analyzing the key instances detected in PAP-QMNIST data, we reveal the tendency of MIL approach to focus on instances with certain features not relevant for malignancy.

To facilitate reproducibility, we share the complete implementation and evaluation framework as open source.
`https://github.com/MIDA-group/OralCancerMILvsSIL`.

Background and related work

With our main interest in the development of an efficient and reliable automated system for OC detection, we investigate existing weakly supervised methods which are applicable also in cases with a limited amount of labeled data. This is stated as one of the remaining challenges in computational pathology by Lu *et al.* [7].

Our previous work [2] indicates that it is possible to distinguish cells from healthy patients and cells coming from OC patients, using only per-patient annotations. This method is based on single instance-level training of deep CNN and we use it as a reference method in our study. The same strategy is evaluated in recent studies, for example in Li *et al.* [8], where it is referred to as "patch-based without considering MIL". In our current study, we explore this approach further, to understand whether it can help to find cells that are highly relevant for OC diagnosis.

There are other methods that can be relevant for our problem. They lie on the intersection of two research directions: (i) weak supervision, due to the absence of cell-level annotations, and (ii) interpretability, providing cell-level information to reach trustworthy solutions and reliable utilization. The approaches that can meet both these requests and at the same time perform well for WSI analysis are: attention-based deep MIL (ABMIL) [9], a method by Campanella *et al.* [10], dual-stream MIL (DSMIL) [8], clustering-constrained-attention MIL (CLAM) [7], and a more recent transformer-based MIL (TransMIL) [11]. The work on ABMIL proposes to incorporate attention mechanisms (and by that interpretability) to MIL, to provide insight into the contribution of each instance. The method by Campanella *et al.* involves aggregation for MIL based on recurrent neural network (RNN). DSMIL is a fusion of a novel non-local attention-based pooling operator with self-supervised contrastive learning and multiscale pyramidal scheme to extract representations. CLAM is an attention-based method with instance-level clustering. TransMIL is designed using Transformer [12] and considers correlation among instances in a bag. With our current focus on evaluating feasibility of using weakly supervised learning methods on our OC data from low number of patients, with large number of cells for some patients, and unbalanced number of cells among patients, not all above mentioned methods are equally applicable. Some of the algorithms are partly tailored specifically for histology, and not cytology data analysis; they consider spatial relation among instances (cells), which is not relevant for the rather randomly distributed cells on cytology WSIs. Such are the introduction of RNN for aggregation in Campanella *et al.* [10], the multiscale approach in Li *et al.* [8] and the pyramid position encoding generator in TransMIL [11].

In this study, along with conventional (single instance) DL approach involving training per patch using weak labels, we consider a frequently used representative of MIL methods – ABMIL – which is observed to perform well on similar tasks. This method both demonstrates good performance at a bag level and also offers interpretability at an instance level. ABMIL is an end-to-end method, and so is the SIL approach we consider; we believe that end-to-end approaches can learn features that represent data better than features obtained in non-end-to-end manner (*e.g.* engineered or pretrained). However, ABMIL cannot be applied directly on bags with very large number of instances (such as, *e.g.*, WSI in cytology, which may have more than 100,000 cells per slide) due to GPU memory constraints. Other MIL approaches tackle this problem using, for example, self-supervision [8], selection of instances in a bag during training [10] or features pretrained on ImageNet [7, 11]. In a previous study [13], we observed that ABMIL can work well with within-bag sampling, benefiting from end-to-end learning, while overcoming memory requirements imposed by WSI. Therefore, in this study we evaluate ABMIL with sampling, following [13], on OC data and synthetic PAP-QMNIST data.

Fig 1. Examples of images from datasets. Top: OC; Bottom: PAP-QMNIST. Images of the digit "4" constitute key instances in the PAP-QMNIST dataset and appear only in positive bags (an example is the third image in (c)), whereas images of other digits appear in both positive and negative bags.

Comparative evaluation

Considered single instance learning – SIL – method

We follow the weakly supervised approach proposed in Lu *et al.* [2], *i.e.*, we use CNN to classify cells (instances), labeled as the patient (bag) they come from, into two classes, healthy and cancer. To select a model for inference, we observe the performance on a validation set; we choose to use F1 score, suitable in presence of the class imbalance in terms of the number of instances. We predict the bag label by aggregating the instance predictions for each bag; if a bag contains a percentage of positively classified instances which is higher than a threshold t, then the bag is assigned a positive label.

Considered multiple instance learning – MIL – method

We use a modified version of ABMIL, Ilse *et al.* [9], with modifications as proposed in Koriakina *et al.* [13]; instead of using original bags (non-feasible for the OC dataset due to the very large bags, containing all cells in a WSI), we use smaller mini-bags sampled from the original bags. Our previous study indicated an increase in Area under the ROC Curve (AUC) at the instance level for smaller mini-bags, as long as there is a sufficient (dataset dependent) number of key instances per mini-bag to be detected [13]. Due to the balanced number of bags (patients) for the two classes in our data, we may directly rely on bag classification accuracy for selecting a model for inference.

Data

Oral Cancer Dataset

The OC Dataset consists of liquid-based (LBC) prepared PAP-stained slides of brush-sampled cells from the oral cavity of 24 patients. Slides were imaged using a NanoZoomer S60 slide scanner, 40×, 0.75 NA objective, at 11 z-offsets (stepping 0.4 µm) providing RGB WSIs of size 103,936 × 107,520 × 3, 0.23 µm/pixel.

The method described in Lu *et al.* [2] was used to detect cell nuclei for which 80×80 pixel regions were cut out, each with one centered nucleus in focus. This provides in total 196,243 cells from healthy and 111,596 cells from OC patients. Example images are shown in Fig. 1(a) and (b). The patient-level diagnoses are set based on histological tissue sample analysis (used as ground truth), where "positive" labels are associated with patients with a detected malignancy. Among 24 patients in the OC dataset, there are 12 patients with histologically confirmed malignancy and 12 healthy patients. To assign a reliable cell-level annotation is not feasible; it is simply a too difficult task to perform even by a highly skilled cytotechnologist.

The study was performed in compliance with the Declaration of Helsinki and approved by the Ethical Review Board Stockholm Sweden (2015-1213-31 and 2019-00349). Informed written consent was obtained from all participants. The study does not involve minors. Data were accessed for research purposes during 2020 and 2021.

PAP-QMNIST Dataset

Reliable cell-level annotations in OC data are scarce. That makes it difficult to judge the outcome of experiments, *e.g.* whether the dataset is too challenging to be processed by a particular method or the parameters of the method are not tuned properly. To facilitate method development and evaluation, we introduce a new synthetic dataset, PAP-QMNIST (S1 Dataset), which is created as a proxy for OC data, while having reliable ground truth also at the instance (cell) level. To facilitate clear interpretation of our studies, we intentionally chose to utilize objects which are easy to recognize (digits) and where there is no hesitation on which properties are important; the shape is the important property for the PAP-QMNIST classes, while color or rotation, for example, are not.

Previously, MNIST-bags [9], CIFAR10-bags [14], QMNIST-bags [13], and Imagenette-bags [13] have been used for development and evaluation of MIL approaches. These datasets are not well suited to serve as a proxy model of our OC Dataset; they are not of appropriate size (they are too small) and do not provide enough images per bag (patient). PAP-QMNIST (see Fig. 1(c) and (d)) is created taking these issues into account. We base PAP-QMNIST on the QMNIST dataset [15], for which the object (digit) is located in the central part of the image, similarly to (the detected and cut-out) nuclei in our OC Dataset, and where the large number of images enables us to create a larger dataset than above mentioned existing datasets for MIL development.

We rescale original QMNIST images to the size of images in the OC Dataset using bilinear interpolation, we add color and augment this dataset by including images transformed by transformations expected in OC data to replicate the number of patients and number of images per patient in the OC Dataset. The colorization of resized QMNIST images is performed as follows: from a sample of 5280 images from the OC dataset, we plot the histogram of each color channel and approximate them by a normal distribution for the green and blue channels and a skewed normal distribution for the red channel. We then sample color values from these distributions for each color channel while avoiding values which, in combination with the other two channels, give colorization visually far from colors of OC data. The same colorization approach is applied for positive and negative classes and the colorization does not affect the class label. This *a priori* knowledge that color is not a relevant feature is useful when evaluating the decision making of the trained networks; if color appears as an important feature, it is an indication that the network probably has over-fitted the training data.

Cell images in the OC dataset are not oriented, since cells (as opposed to digits) may appear in any rotation. Therefore, in the creation of PAP-QMNIST we rotate digits by a random angle. Additionally, we add horizontal and vertical flips, as well as Gaussian noise, blur, motion blur, random fog and random change in brightness and contrast [16].

The original QMNIST dataset is composed of train and test sets, each with 60k images. We randomly extract 20% of the original QMNIST test set to form a validation set while the remaining 80% are kept as test set. PAP-QMNIST train, validation, and test sets are created from the correspondingly sampled train, validation, and test sets of such split QMNIST.

In our experiments, we are evaluating the effect of different ratios of key instances in the data on the success of automated detection and interpretation. This corresponds to evaluation of the impact of the ratio of malignant cells (responsible for the diagnosis) and the total number of cells in a bag, on the correctly made diagnosis. When creating PAP-QMNIST experimental data, we can define the number of key instances in a bag (patient sample). Ideally, this number should mimic the number of key instances for a patient in the OC dataset. However, it is virtually impossible to estimate the percentage of key instances in the OC dataset. Therefore, we make experiments with different versions of PAP-QMNIST. First we assume an ideal case with the same percentage of

Fig 2. Used beta distributions, defining the fraction of key instances in positive PAP-QMNIST bags.

key instances for all patients and we set the percentage of key instances for each bag to 5, 10, 20, or 30. Second, to introduce variations that could be observed in real data, we design experiments with PAP-QMNIST where the percentage of key instances varies in positive bags and is sampled according to a beta distribution (commonly considered a suitable model for the random behavior of percentages and proportions) with the mean of 17.5% (the middle of the range $[5 - 30\%]$) and standard deviations of 5% and 10% (Fig. 2). These datasets are referred to as *PAP-QMNIST-beta1* and *PAP-QMNIST-beta2* respectively. Parameters α and β for beta distribution with standard deviation of 5% are respectively equal to 9.932 and 46.82, and for beta distribution with standard deviation of 10% are 2.352 and 11.086 respectively. We choose the digit "4" to represent key instances in the PAP-QMNIST data; this provides a suitably difficult problem, where rotated versions of this digit may look very similar to several other digits in the data set. The key instances are present *only* within positive bags, *i.e.*, those representing samples from patients with malignancy.

Experimental setup

All the versions of PAP-QMNIST dataset, with different percentages of key instances in positive bags, are first generated as MIL datasets, *i.e.*, images are sampled into bags; after that, the SIL format of the versions is created by assigning positive labels to all images from positive bags while all images from negative bags are assigned negative labels. The OC Dataset is intrinsically a MIL dataset, since it is divided into bags that correspond to patients. The SIL format of the OC Dataset is created in the same way as the SIL format of PAP-QMNIST dataset. 24 bags in folds of PAP-QMNIST are sampled once (for each key instance setting) and then permuted for different folds (in the same way as bags of the OC Dataset).

We use 9-fold cross-validation for all methods and for both PAP-QMNIST and OC Dataset, where data is separated on the bag (patient) level. For each fold 6 negative bags and 6 positive bags are used for training, 2 negative bags and 2 positive bags for validation, and 4 negative bags and 4 positive bags for testing. It always holds that in the test set there are no instances from bags used in training or validation. Each bag appears in the test set of three folds with different combinations of bags for training, validation and testing.

All experiments are performed using PyTorch [17]. The Albumentations [16] package is used to augment both PAP-QMNIST and OC Dataset during training, and PAP-QMNIST during creation. Augmentations used during training are horizontal and vertical flips, rotation by multiples of 90 degrees (thereby avoiding interpolation), added Gaussian noise. Images are standardized by the mean and standard deviation of the intensities in each of the three color channels over the whole dataset, for both PAP-QMNIST and OC Dataset. For both considered methods (ABMIL with sampling and SIL), and both datasets (PAP-QMNIST and OC Dataset), we evaluate three different architectures: LeNet architecture as in Ilse *et al.* [9], ResNet18 [18] and SqueezeNet [19]. The choice of these architectures is made considering the size of images and their preliminary performance on PAP-QMNIST data. Learning rates and weight decay regularisation coefficients were selected for different architectures based on optimization on a subset of PAP-QMNIST (see S1 Table 5).

During training, we store the model weights for each epoch. To finally select the model to use at inference, we observe the moving average (over a window of 15 epochs) of F1 score for SIL, and classification error for ABMIL with sampling, on the validation

set. For the moving average window with the highest average F1 score or lowest classification error (for SIL and ABMIL with sampling, respectively), we select the model which provides the highest validation F1 score/the lowest classification error within that window. We select this model after initial training for 20 epochs (for both SIL and ABMIL with sampling) to avoid possible fluctuations at the beginning of training.

For SIL, we set mini-batch size to 56 images and the maximum number of training epochs to 150 for both PAP-QMNIST and OC Dataset, which is defined beforehand, observing that the convergence to a maximum F1 score on the validation data is reached earlier than this number of epochs. A more detailed analysis on the effect of mini-batch sizes on the performance of SIL can be found in S1 Appendix.

For ABMIL with sampling, we perform experiments with mini-bag sizes with 2500, 1200, and 500 instances, corresponding to 26.9%, 12.9%, and 5.4% of the average number of instances per bag. We use a model parallel technique, sending different parts of the model to two different GPUs, each of 12, 16, or 32 GB of VRAM, depending on the mini-bag size and network architecture. The approximate number of test evaluations for each instance (see Koriakina *et al.* [13]) is calculated based on the bag with the highest number of instances, for which we make approximately 10 test evaluations per instance. We set the maximum number of training epochs to 1500, having observed beforehand that the best validation performance is reached well before this number.

Quantitative evaluation

We perform quantitative evaluation of the observed algorithms by computing:

1a, accuracy at the bag ("patient") level for PAP-QMNIST;

1b, recall at the instance ("cell") level, $Recall@K_i$ (a.k.a. crossover point of precision and recall), for the instances with the K_i top attention weights/prediction scores, where K_i is the number of key instances in a bag i for PAP-QMNIST;

2, accuracy at the patient (bag) level for OC dataset.

In all cases, we observe different bag sizes for ABMIL with sampling and different ratios of key instances in the bags.

For ABMIL with sampling, we calculate accuracy at the bag (patient) level in the same manner as in Koriakina *et al.* [13], by computing a majority voted mini-bag label for each test bag. Metric at the instance level is calculated only for the positive (containing images of the digit "4") bags in the test set of PAP-QMNIST, following [13]. Due to sampling, we aggregate an average attention weight for each instance. First, we store attention weights for an instance from all mini-bags where this instance is present and then compute its average attention weight using only attention weights from mini-bags with the same predicted bag label as the majority bag label for all mini-bags where this instance is present. The attention weights are normalized for each mini-bag to the range [0,1]. An instance in bag i is considered to be a correctly detected key instance when all of the following holds: (i) the predicted bag label is positive, (ii) the true instance label is positive, and (iii) the value for the average attention weight for the instance falls among top K_i average attention weights of the bag i, where K_i is the number of key instances in bag i.

To make the comparison between ABMIL with sampling and SIL as fair as possible, we compute performance metrics for SIL in the same way as we compute them for ABMIL: first, we obtain bag level metric and then use bag labels together with instance labels to compute instance level metric. The threshold t, separating positive and negative bags is chosen as the average of thresholds from the 9 validation folds, where each such threshold is computed as the middle value between the percentage of images classified as positive (softmax score>0.5) in positive and negative bags. For PAP-QMNIST with beta-distributed percentage of key instances in positive bags and

OC Dataset we use the median threshold over 9 validation folds instead of the average threshold, to reduce the possible influence of threshold values of folds where a combination of validation bags results in a threshold far from the rest of the folds.

We then compute SIL instance-level metric for the PAP-QMNIST test set by using prediction scores from the softmax layer. Due to the different nature of ABMIL with sampling and SIL, bags classified as negative by SIL may contain instances which are classified as positive, whereas bags classified as negative by ABMIL with sampling may not, by definition, contain any positive (*i.e.* key) instances [9]. For a fair methods comparison, we compute key instance detection performance of SIL in a similar way as for ABMIL with sampling, *i.e.*, taking into account only the instances coming from bags with a true positive label. An instance in bag i is considered to be a correctly detected key instance when all of the following holds: (i) the predicted bag label is positive, (ii) the true instance label is positive, and (iii) the softmax score for the instance falls in top K_i scores of that bag i, where K_i is the number of key instances in bag i. (Note that a key instance (softmax among top K_i in a positively predicted bag) is not the same as an above-mentioned instance classified as positive (softmax score>0.5).)

Qualitative evaluation

In addition to quantitative evaluation, we perform a visual inspection of top ranked key instances detected in PAP-QMNIST and OC Datasets by both considered methods. We observe instances with the highest attention weights/prediction scores for each correctly detected positive bag (actually positive and also detected as positive (TP)) by the considered methods, trained and evaluated on the same data. We analyze the top 25 such instances detected in one of the folds of all generated versions of the PAP-QMNIST dataset.

Qualitative evaluation of results on the OC Dataset is performed with the participation of an expert cytotechnologist. Our aim is to identify if there exists a correspondence between the key instances identified by the automated system (based on each of the models) and those identified by a human expert. The cytotechnologist is presented with, for each patient in the test set, the 36 instances with the highest attention weights/prediction scores, as identified by the system in three folds of the OC data, and is asked to identify images/cells with characteristics which can be related to malignancy. Cytological annotation is performed according to the Bethesda system [20, 21], where ASC-US stands for 'atypical squamous cells of unknown significance' and ASC-H for 'atypical squamous cells, cannot exclude a high-grade lesion'.

The images shown to our expert cytotechnologist are cut-outs of a larger size, 170×170 pixels, instead of the 80×80 pixels used by the methods, to make the examination more reliable (since such a setup is more similar to what cytotechnologists typically use). This difference does not affect the study, since we do not compare human performance to the performance of the considered methods, but we are interested in obtaining the opinion of an expert on the relevance of the cells the methods report as being most related to the malignant class.

Results

PAP-QMNIST dataset

We show accuracy at the bag level and $Recall@K_i$ at the instance level for all considered methods in Fig. 3 and present mean and standard deviation of metrics in Tables 1 and 2. On average, for PAP-QMNIST, both metrics have higher values with 10%, 20% and

Fig 3. Test set performance for ABMIL with sampling and SIL on PAP-QMNIST. Top: Accuracy at the bag level. Bottom: Recall at the instance level, *Recall@*K_i, for instances with the K_i top attention weights/prediction scores, where K_i is the number of key instances in bag i. Three mini-bag sizes, 500, 1200 and 2500, used for ABMIL with sampling are indicated on the x-axis. Larger values of accuracy and *Recall@*K_i are better. The box plots display minimum, first quartile, median, third quartile, and maximum (the five-number summary) over 9 folds.

Table 1. Accuracy at the bag level of the ABMIL with sampling and SIL methods on PAP-QMNIST test data. Three mini-bag sizes, 500, 1200 and 2500, used for ABMIL with sampling are indicated on the x-axis. Mean and standard deviation of the metric are computed over 9 folds. Bold face indicates the best performance for each dataset.

Percentage of key instances in positive bags		ABMIL 500	ABMIL 1200	ABMIL 2500	SIL
5%	LeNet	0.833±0.236	0.722±0.211	0.556±0.168	0.764±0.239
	ResNet	0.528±0.115	0.542±0.102	0.486±0.039	**1.000±0.000**
	SqueezeNet	0.528±0.079	0.500±0.000	0.500±0.000	0.931±0.157
10%	LeNet	0.944±0.157	0.958±0.118	0.972±0.052	0.931±0.157
	ResNet	0.944±0.086	0.806±0.221	0.819±0.168	0.931±0.157
	SqueezeNet	0.569±0.157	0.500±0.000	0.500±0.000	**1.000±0.000**
20%	LeNet	**1.000±0.000**	0.944±0.157	0.986±0.039	0.972±0.052
	ResNet	**1.000±0.000**	0.889±0.208	0.694±0.221	0.986±0.039
	SqueezeNet	0.986±0.039	0.972±0.079	0.861±0.208	0.944±0.157
30%	LeNet	0.986±0.039	**1.000±0.000**	0.972±0.079	0.944±0.157
	ResNet	**1.000±0.000**	0.986±0.039	0.958±0.083	**1.000±0.000**
	SqueezeNet	**1.000±0.000**	**1.000±0.000**	0.944±0.157	**1.000±0.000**
PAP-QMNIST-beta1, beta-distributed mean=17.5%, std=5%	LeNet	**1.000±0.000**	0.944±0.157	0.986±0.039	0.681±0.213
	ResNet	**1.000±0.000**	0.917±0.167	0.819±0.205	**1.000±0.000**
	SqueezeNet	0.944±0.157	0.792±0.236	0.764±0.239	**1.000±0.000**
PAP-QMNIST-beta2, beta-distributed mean=17.5%, std=10%	LeNet	**0.986±0.039**	0.875±0.186	0.931±0.086	0.778±0.227
	ResNet	0.931±0.086	0.903±0.153	0.847±0.193	0.875±0.083
	SqueezeNet	0.931±0.157	0.889±0.161	0.833±0.212	0.889±0.092

30% of key instances per positive bag, than with 5% of key instances per bag, for all the network architectures used. We also observe that the training is more sensitive to hyper-parameter tuning at 5% key instances. For ABMIL with sampling, we observe increased accuracy at the bag level and *Recall@*K_i when the percentage of key instances in positive bags increases. We see that high accuracy at the bag level corresponds to relatively high *Recall@*K_i for this method.

Somewhat unexpectedly, the accuracy and *Recall@*K_i values of the SIL approach are higher or comparable to those of ABMIL with sampling. SIL is able to detect the majority of the key instances for datasets with 10%, 20% and 30% of key instances. Performances of both SIL and ABMIL with sampling on *PAP-QMNIST-beta1* are mainly similar to the performance of PAP-QMNIST with 20% of key instances, except the decreased performance of LeNet-based SIL. Neither of the methods is able to classify all the bags of *PAP-QMNIST-beta2* correctly, however, both methods are able to classify all bags of *PAP-QMNIST-beta1*. The bag-level metric is decreasing for SIL based on LeNet on *PAP-QMNIST-beta1* and *PAP-QMNIST-beta2*. The bag-level metric has lower values for SIL on the *PAP-QMNIST-beta2* as compared to ABMIL.

Overall, the instance-level metric has higher values for SIL on all the datasets as compared to ABMIL with sampling, and on average, SIL outperforms ABMIL with sampling at the bag level for methods based on two out of three architectures.

Examples of instances with the highest attention weights/prediction scores, for PAP-QMNIST dataset with different percentage of key instances, from bags classified as positive by ABMIL with sampling and SIL, are presented in Fig. 4. Test bags from PAP-QMNIST dataset with positive bag labels that are (incorrectly) classified as negative (ABMIL with sampling with mini-bag size of 500 instances on PAP-QMNIST with 5% of key instances and SIL on *PAP-QMNIST-beta2*) are not shown in Fig. 4.

Table 2. *Recall@K$_i$* at instance level of the ABMIL with sampling and SIL methods on PAP-QMNIST test data. Three mini-bag sizes, 500, 1200 and 2500, used for ABMIL with sampling are indicated on the x-axis. Mean and standard deviation of the metric are computed over 9 folds. Bold face indicates the best performance for each dataset.

Percentage of key instances in positive bags		ABMIL 500	ABMIL 1200	ABMIL 2500	SIL
5%	LeNet	0.159±0.122	0.066±0.076	0.036±0.044	0.514±0.280
	ResNet	0.024±0.034	0.025±0.029	0.034±0.029	**0.862±0.037**
	SqueezeNet	0.009±0.017	0.010±0.019	0.012±0.017	0.827±0.047
10%	LeNet	0.332±0.065	0.252±0.030	0.236±0.076	0.836±0.028
	ResNet	0.248±0.069	0.214±0.057	0.180±0.064	**0.936±0.007**
	SqueezeNet	0.084±0.088	0.017±0.031	0.011±0.020	0.931±0.008
20%	LeNet	0.374±0.052	0.335±0.090	0.325±0.082	0.923±0.006
	ResNet	0.403±0.019	0.380±0.055	0.273±0.106	**0.959±0.006**
	SqueezeNet	0.469±0.095	0.455±0.107	0.499±0.150	0.853±0.302
30%	LeNet	0.449±0.047	0.472±0.052	0.485±0.042	0.933±0.025
	ResNet	0.361±0.061	0.332±0.092	0.349±0.099	**0.976±0.002**
	SqueezeNet	0.544±0.047	0.558±0.048	0.567±0.207	0.972±0.004
PAP-QMNIST-beta1,	LeNet	0.401±0.030	0.261±0.106	0.256±0.058	0.748±0.130
beta-distributed	ResNet	0.348±0.036	0.305±0.053	0.260±0.085	0.945±0.005
mean=17.5%, std=5%	SqueezeNet	0.287±0.108	0.225±0.169	0.179±0.144	**0.947±0.005**
PAP-QMNIST-beta2,	LeNet	0.379±0.082	0.300±0.145	0.281±0.078	0.673±0.307
beta-distributed	ResNet	0.337±0.059	0.305±0.045	0.315±0.040	0.724±0.161
mean=17.5%, std=10%	SqueezeNet	0.403±0.062	0.387±0.126	0.286±0.150	**0.748±0.175**

Fig 4. Examples of instances detected by ABMIL with sampling and SIL approaches in the PAP-QMNIST dataset. Top 25 instances with the highest attention weights/prediction scores for each of the four test bags with positive label are shown, detected as positive in one of the folds of the PAP-QMNIST dataset. (a)-(e) ABMIL with sampling based on ResNet18 (similar results observed for other architectures) and with mini-bag size of 500 instances. ABMIL with sampling misclassified the bags with 5% of key instances, therefore this case is not presented (no key instances are detected). Similarly, SIL misclassified one bag on *PAP-QMNIST-beta2*, and examples of instances for that bag are not present. (f)-(k) SIL based on ResNet18. The percent of key instances per bag in the datasets is indicated under each subfigure. Red polygons delineate wrongly identified key instances – digits other than "4".

One can observe that more diverse in color and rotation key instances are detected by SIL as compared to ABMIL with sampling, as well as that a higher number of true key instances, among the instances with the highest attention weights/prediction scores, are found by SIL than by ABMIL with sampling. This observation is in agreement with the values of the instance metric *Recall@K$_i$* for SIL, which exceed the values of *Recall@K$_i$* for ABMIL with sampling on average for all the datasets.

Fig. 5 illustrates the percentage of instances detected by SIL as positive for different architectures and different percentage of key instances per bag. The percent of instances classified as positive roughly corresponds to the true percent of key instances per bag for SqueezeNet and ResNet18-based SIL, matching the high *Recall@K$_i$* at the instance level in Fig. 3 (bottom row). The percentage of instances detected as positive by LeNet-based SIL corresponds worse to the true percent of key instances for versions of PAP-QMNIST dataset with fixed percentage of key instances and differs a lot from the true percent of key instances for *PAP-QMNIST-beta1* and *PAP-QMNIST-beta2*.

Oral Cancer Dataset

Accuracy at the bag level for OC Dataset is shown in Fig. 6, where accuracies of methods based on considered architectures, on average, are all above 0.75 (see Table 3). The accuracy of SIL is higher than the accuracy of ABMIL with sampling. The average accuracy of SIL exceeds 0.9 for all three architectures. The best-performing architecture, ResNet18, reaches a patient-level accuracy of 94.4%, which exceeds reported human

Fig 5. Percentage of instances identified as positive by SIL approach on PAP-QMNIST dataset. Instances identified as positive by SIL approach have softmax score>0.5. Percentage is shown per each test bag for versions of PAP-QMNIST dataset with different key instance ratios. Standard deviation is indicated by error bars and calculated for the 3 different folds in which each bag appears. Bags 0-11 are negative, bags 12-23 are positive.

Fig 6. Accuracy at the bag level of the ABMIL with sampling and SIL methods on OC Dataset. Accuracy is shown for the test set of OC Dataset. Three mini-bag sizes, 500, 1200 and 2500, used for ABMIL with sampling are indicated on the x-axis. The box plots display minimum, first quartile, median, third quartile, and maximum (the five-number summary) over 9 folds.

Table 3. Accuracy at the bag level of the ABMIL with sampling and SIL methods on OC Dataset.

	ABMIL 500	ABMIL 1200	ABMIL 2500	SIL
LeNet	0.847±0.174	0.847±0.163	0.806±0.178	0.917±0.088
ResNet	0.844±0.111	0.766±0.206	0.844±0.130	**0.944±0.091**
SqueezeNet	0.778±0.174	0.861±0.171	0.806±0.235	0.917±0.088

Accuracy is shown for the test set of OC Dataset. Mean and standard deviation of the metric are computed over 9 folds. Bold face indicates the best performance.

experts' performance on this type of tasks and therefore shows great promise for future clinical use. The percentage of cells identified by SIL as coming from patients with OC for each of the 24 slides, is illustrated in Fig. 7.

Figure 8 shows four mosaics of 36 cells each, in a form presented to the cytotechnologist. We prepared 118 such mosaics (see Table 4). Each selection of 36 cells contains the instances (out of, on average, 9,300 cells per patient) with the highest attention weights/prediction scores from the bags predicted as positive by the methods. The subsequent evaluation performed by the cytotechnologist resulted in 94 of these mosaics labeled as containing no suspicious cells but only cells from the categories: normal superficial cells (see Fig. 8(a)), a mixture of normal superficial cells and normal intermediary cells, normal intermediary cells, or a mixture of normal superficial cells and cell debris or blood cells such as lymphocytes and neutrophils; 21 of the sets were annotated as benign but with reactive changes, (Fig. 8(b)) or sets that contained a minority of not normal cells, such as metaplastic, ASC-US, or ASC-H, with remaining cells being normal. Three of the sets were annotated as suspicious for high grade dysplasia (see Fig. 8(c)), all coming from one patient and detected by SIL with all three architectures. For reference, a set of cells predicted by SIL approach as negative and annotated as normal cells are shown in Fig. 8(d). For ABMIL with sampling, out of the mosaics with the top 36 detected key instances from bags predicted as positive, 21% contained cells annotated as reactive or not normal (for ABMIL with mini-bag size of 2500 instances - the best performing mini-bag size at the qualitative evaluation on OC Dataset), while out of the mosaics with key instances detected using SIL, 28% contained cells annotated as reactive or not normal.

Discussion

The theory of MIL defines that one key instance in a bag makes the bag positive, however, we observe when training on PAP-QMNIST that one key instance is not enough for ABMIL with sampling to classify bags reliably, but rather some percentage (in our experiments, 10% or more) of key instances per positive bag is required. The used performance measures for ABMIL, reach higher values for mini-bags of size 500 instances, on average, compared to ABMIL with larger mini-bags, on both PAP-QMNIST and OC Datasets. This is in agreement with the previous study by

Fig 7. Percentage of instances (cells) identified as positive by SIL approach on the OC Dataset. Instances identified as positive by SIL approach have softmax score>0.5. Percentage is shown per each test bag (patient). Error bars indicate standard deviation over the 3 different folds in which each bag appears. The black dashed line is the threshold computed based on validation set over 9 folds for each architecture type. Bags 0-11 (blue) are negative, the rest (yellow) are positive.

Fig 8. Examples of instances detected by ABMIL with sampling and SIL approaches in the OC Dataset. Top 36 instances with the highest attention weights/prediction scores on test set of the OC Dataset are shown, together with the corresponding annotations by cytotechnologist.

Koriakina *et al.* [13].

The SIL approach, on average, outperform ABMIL with sampling on versions of PAP-QMNIST dataset with a fixed percentage of key instances at both the bag and instance levels, with ResNet18 and SqueezeNet, reaching high values of accuracy and $Recall@K_i$ also for datasets with (only) 5% of key instances. To consider variation in the percentage of key instances that can be present in real data, we create PAP-QMNIST datasets where the percentage of key instances in positive bags is drawn according to beta distributions. The performance of LeNet-based SIL on PAP-QMNIST with beta-distributed percentage of key instances does not reach the performance of ResNet18 and SqueezeNet-based SIL, presumably due to the shallower architecture.

Accuracy at the bag level, averaged over 9 folds, of the different methods on real cytological OC Dataset is lower than accuracy at the bag level on the synthetic PAP-QMNIST, however, we show that it is possible to classify nearly all bags correctly also in OC Dataset.

Studying the top key instances detected by ABMIL with sampling in the PAP-QMNIST dataset (Fig. 4) indicates that ABMIL with sampling can be prone to paying attention to color and orientation, features which, for this dataset, are known (by design) to not be relevant. Based on our results we arrive at the conclusion that ABMIL with sampling has a tendency to focus on a subset of the present key instances (having the same shape, color, or orientation), whereas the SIL approach shows to be less prone to this type of mode collapse.

Qualitative evaluation of the methods on OC Dataset at the instance level is performed involving an expert. The majority of sets of instances with the highest attention weights/prediction scores from the bags predicted as positive by the methods are annotated by the cytotechnologist as normal superficial cells or cells/debris not related to malignancy. PAP-stained superficial cells have a distinct orange/pink color and are not specific for malignancy but can be present both for healthy patients and for patients with OC. A possible explanation for why such cells are predicted as key instances is the fact that they do appear more frequently within patients with

Table 4. Qualitative evaluation of ABMIL with sampling and SIL methods on OC Dataset. Agreement of methods' prediction with judgements made by cytotechnologist on the top ranked instances. See text for details.

	Predicted bag class for evaluated mosaics		Mosaics containing suspicious cells according to cytotechnologist	
	negative	positive	fraction	percent
ABMIL 500	11	25	3/25	12%
ABMIL 1200	7	29	5/29	17%
ABMIL 2500	8	28	6/28	21%
SIL	0	36	10/36	28%
Total number	26	118		

malignancy than within healthy patients for our collected OC Dataset; in that sense, the models have correctly found a relation, however, the medical expertise finds this relation as not trustworthy for a decision. This highlights the importance of interpretable approaches, in focus of our study. Among the sets of predicted key instances there are also many cells which, although not indicating clear malignancy, do exhibit some deviation from normality. Such cells are detected by both ABMIL with sampling and SIL, and might be relevant for early detection of OC, *e.g.* suggesting further medical investigation. The SIL approach succeeded to highly rank cells annotated as *high grade dysplasia*, indicating the feasibility of inferring cell-level information from patient-level annotations. These, for a diagnosis of highly relevant cells, were not ranked among the top 36 key instances using ABMIL with sampling for the same annotated data. Note that we do not consider the visual inspection of only 36 cells out of ca 10,000 cells on a whole slide to correspond to any envisioned realistic assessment, but it is a means for us to compare the considered methods.

Considering that a human expert performs labeling of the cells using larger cut-outs than those used by the automated approaches, one may argue that the evaluated methods could also be applied to larger cut-outs of the original image. Classification of larger crops is computationally more demanding, especially for the ABMIL with sampling method, and, more importantly, larger crops contain bigger parts of neighboring cells, which shifts the focus from a particular cell of interest. The cytotechnologist can distinguish clutter from cells and is explicitly focusing on the central one, whereas computer methods may take additional clutter as a feature, which leads to reduced interpretability of the results. We do not see any reason to believe that a change to larger cut-outs would change the relative performance of the observed methods.

Our results indicate that SIL outperforms ABMIL with sampling. This stands in contrast to a common assumption that MIL is more suitable than SIL for learning from weakly annotated data. Possibly our results are due to the nature of the here addressed task, with rather few and very large bags of varying sizes.

We would like to mention that this study is tailored for classification of cytology data, where there is no spatial relation among cells and we make a choice of specific MIL and SIL methods accordingly. The classification of spatially related data may benefit more from methods which consider such relation.

Summary and Conclusion

In this study, we investigate two different weakly supervised DL methods with the aim to reach reliable and interpretable OC detection: ABMIL with within-bags sampling, an approach that belongs to the MIL family of methods, and a more conventional SIL classification. We evaluate performance on PAP-QMNIST – a synthetic dataset mimicking, in terms of several parameters relevant to our analysis, our OC cytological data, as well as on real OC data from 24 patients. On the synthetic PAP-QMNIST data, we explore how the percentage and distribution of key instances in positive bags affect the classification performance.

An overall conclusion of the performed comparative study is that, for this task, we do not observe any reasons to use ABMIL with sampling instead of the less complex and less memory-demanding SIL approach. The exception would be datasets with largely varying percentages of key instances in positive bags, where ABMIL with sampling could outperform SIL on bag level classification. Nevertheless, SIL's ability to detect key instances surpasses, even on such datasets, the ability of ABMIL with sampling, suggesting that cytotechnologists could gain from both methods on such data.

Through the performed experiments, we demonstrate that:

- It is possible not only to separate healthy patients from patients with malignancy, but also to detect cells related to malignancy, by utilizing only patient-level annotations on a fairly small number of patients;
- SIL, on average, outperforms a here observed representative of MIL methods – ABMIL with sampling. Obtained results are consistent on the two datasets, the real cytology data (OC Dataset), and the synthetic PAP-QMNIST dataset;
- The PAP-QMNIST dataset facilitated evaluation of instance-level performance, as well as analysis of interpretability of the methods, and allowed to observe a tendency of ABMIL with sampling to focus on, for the task, not relevant features.

The performed study is a valuable step towards development of interpretable AI-based OC detection methods. Furthermore, we see a potential of the created synthetic PAP-QMNIST dataset for usage in other similar evaluation scenarios.

References

1. Sukegawa S, et al. Clinical study on primary screening of oral cancer and precancerous lesions by oral cytology. Diagnostic Pathology. 2020;15(1):1–6. doi:10.1186/s13000-020-01027-6.
2. Lu J, Sladoje N, Stark CR, Ramqvist ED, Hirsch JM, Lindblad J. A deep learning based pipeline for efficient oral cancer screening on whole slide images. In: International Conference on Image Analysis and Recognition. Springer; 2020. p. 249–261.
3. Conceição T, Braga C, Rosado L, Vasconcelos MJM. A review of computational methods for cervical cells segmentation and abnormality classification. International journal of molecular sciences. 2019;20(20):5114. doi:10.3390/ijms20205114.
4. Cheplygina V, de Bruijne M, Pluim JPW. Not-so-supervised: A survey of semi-supervised, multi-instance, and transfer learning in medical image analysis. Medical Image Analysis. 2019;54:280–296. doi:10.1016/j.media.2019.03.009.
5. Jiang H, Zhou Y, Lin Y, Chan RC, Liu J, Chen H. Deep learning for computational cytology: A survey. Medical Image Analysis. 2022; p. 102691. doi:10.1016/j.media.2022.102691.
6. Quellec G, Cazuguel G, Cochener B, Lamard M. Multiple-instance learning for medical image and video analysis. IEEE reviews in biomedical engineering. 2017;10:213–234. doi:10.1109/RBME.2017.2651164.
7. Lu MY, Williamson DF, Chen TY, Chen RJ, Barbieri M, Mahmood F. Data-efficient and weakly supervised computational pathology on whole-slide images. Nature Biomedical Engineering. 2021;5(6):555–570. doi:10.1038/s41551-020-00682-w.
8. Li B, Li Y, Eliceiri KW. Dual-stream multiple instance learning network for whole slide image classification with self-supervised contrastive learning. In: Proceedings of the IEEE/CVF Conference on Computer Vision and Pattern Recognition; 2021. p. 14318–14328.
9. Ilse M, Tomczak J, Welling M. Attention-based deep multiple instance learning. In: International conference on machine learning. PMLR; 2018. p. 2127–2136.

10. Campanella G, Hanna MG, Geneslaw L, Miraflor A, Silva VWK, Busam KJ, et al. Clinical-grade computational pathology using weakly supervised deep learning on whole slide images. Nature medicine. 2019;25(8):1301–1309. doi:10.1038/s41591-019-0508-1.

11. Shao Z, Bian H, Chen Y, Wang Y, Zhang J, Ji X, et al. Transmil: Transformer based correlated multiple instance learning for whole slide image classification. Advances in Neural Information Processing Systems. 2021;34.

12. Vaswani A, Shazeer N, Parmar N, Uszkoreit J, Jones L, Gomez AN, et al. Attention is all you need. In: Advances in neural information processing systems; 2017. p. 5998–6008.

13. Koriakina N, Sladoje N, Lindblad J. The Effect of Within-Bag Sampling on End-to-End Multiple Instance Learning. In: 2021 12th International Symposium on Image and Signal Processing and Analysis (ISPA). IEEE; 2021. p. 183–188.

14. Seibold C, Kleesiek J, Schlemmer HP, Stiefelhagen R. Self-Guided Multiple Instance Learning for Weakly Supervised Thoracic DiseaseClassification and Localizationin Chest Radiographs. In: Proceedings of the Asian Conference on Computer Vision; 2020.

15. Yadav C, Bottou L. Cold Case: The Lost MNIST Digits. In: Wallach H, Larochelle H, Beygelzimer A, d'Alché-Buc F, Fox E, Garnett R, editors. Advances in Neural Information Processing Systems. vol. 32. Curran Associates, Inc.; 2019.Available from: `https://proceedings.neurips.cc/paper/2019/file/51c68dc084cb0b8467eafad1330bce66-Paper.pdf`.

16. Buslaev A, et al. Albumentations: Fast and Flexible Image Augmentations. Information. 2020;11(2). doi:10.3390/info11020125.

17. Paszke A, Gross S, Massa F, Lerer A, Bradbury J, Chanan G, et al. Pytorch: An imperative style, high-performance deep learning library. Advances in neural information processing systems. 2019;32:8026–8037.

18. He K, Zhang X, Ren S, Sun J. Deep residual learning for image recognition. In: Proceedings of the IEEE conference on computer vision and pattern recognition; 2016. p. 770–778.

19. Iandola FN, Han S, Moskewicz MW, Ashraf K, Dally WJ, Keutzer K. SqueezeNet: AlexNet-level accuracy with 50x fewer parameters and¡ 0.5 MB model size. arXiv preprint arXiv:160207360. 2016;.

20. Nayar R, Wilbur DC. The Bethesda system for reporting cervical cytology: a historical perspective. Acta cytologica. 2017;61(4-5):359–372. doi:10.1159/000477556.

21. Sivakumar N, Narwal A, Kumar S, Kamboj M, Devi A, Pandiar D, et al. Application of the Bethesda system of reporting for cervical cytology to evaluate human papilloma virus induced changes in oral leukoplakia, oral squamous cell carcinoma, and oropharyngeal squamous cell carcinoma: A cytomorphological and genetic study. Diagnostic Cytopathology. 2021;49(9):1036–1044. doi:10.1002/dc.24813.

Fig 9. The performance of SIL trained for 1500 epochs on test set of the PAP-QMNIST. Four mini-batch sizes are indicated on x-axis. The box plots display minimum, first quartile, median, third quartile, and maximum (the five-number summary) over 9 folds. **Top:** Accuracy at the bag level. **Bottom:** Recall at the instance level, *Recall@*K_i, for instances with the K_i top attention weights/prediction scores. K_i is the number of key instances in a bag i.

Supporting information

S1 Table. The used optimization parameters for ABMIL with sampling and SIL for different neural network architectures

Table 5. The used optimization parameters for ABMIL with sampling and SIL for different neural network architectures.

	Learning rate		Weight decay regularisation coefficient	
	ABMIL	SIL	ABMIL	SIL
LeNet	$5 \cdot 10^{-5}$	10^{-4}	10^{-6}	10^{-4}
ResNet	$5 \cdot 10^{-6}$	10^{-4}	10^{-5}	10^{-6}
SqueezeNet	$5 \cdot 10^{-5}$	10^{-4}	10^{-5}	10^{-6}

S1 Appendix. The effect of mini-batch size on the performance of SIL observed on PAP-QMNIST: To ensure an appropriate choice of mini-batch size, we have evaluated SIL performance for mini-batch sizes of 56, 500, 1200 and 2500 images, allowing the model to be trained for 1500 epochs instead of 150 epochs and with the same stopping criteria. The increased number of epochs for training was chosen due to the observation that models trained with larger mini-batch sizes were converging slower than models trained with a mini-batch size of 56. We observe (S1 Fig. 9) that the performance does not differ a lot depending on the mini-batch size, and there is no visible trend. Interestingly, for the models trained with mini-batches of 56 images, there are several convergence plateaus for the validation set where one may select to pick a model for inference, however, the earlier solution (found within the first 150 epochs) generalizes better on the test sets (early stopping). Considering the additional required computational resources for training with larger mini-batch sizes, along with a better performance from early stopping, we conclude that mini-batch size of 56 images and the overall number of training epochs equal to 150 for SIL are most suitable.

S1 Dataset. PAP-QMNIST dataset. The PAP-QMNIST datasets generated and analysed during the current study are available in the Zenodo repository, `https://doi.org/10.5281/zenodo.7020312`, together with the code for generating PAP-QMNIST data.

Acknowledgements

This work is supported by: Sweden's Innovation Agency (VINNOVA), grants 2017-02447 and 2020-03611, and the Swedish Research Council, grant 2017-04385. A part of the computations was enabled by resources provided by the National Academic Infrastructure for Supercomputing in Sweden (NAISS) and the Swedish National Infrastructure for Computing (SNIC) at Chalmers Centre for Computational Science and Engineering (C3SE), partially funded by the Swedish Research Council through grant agreements no. 2022-06725 and no. 2018-05973.

Competing interests

The authors declare no competing interests.

Figure

Click here to access/download;Figure;Fig1.tiff

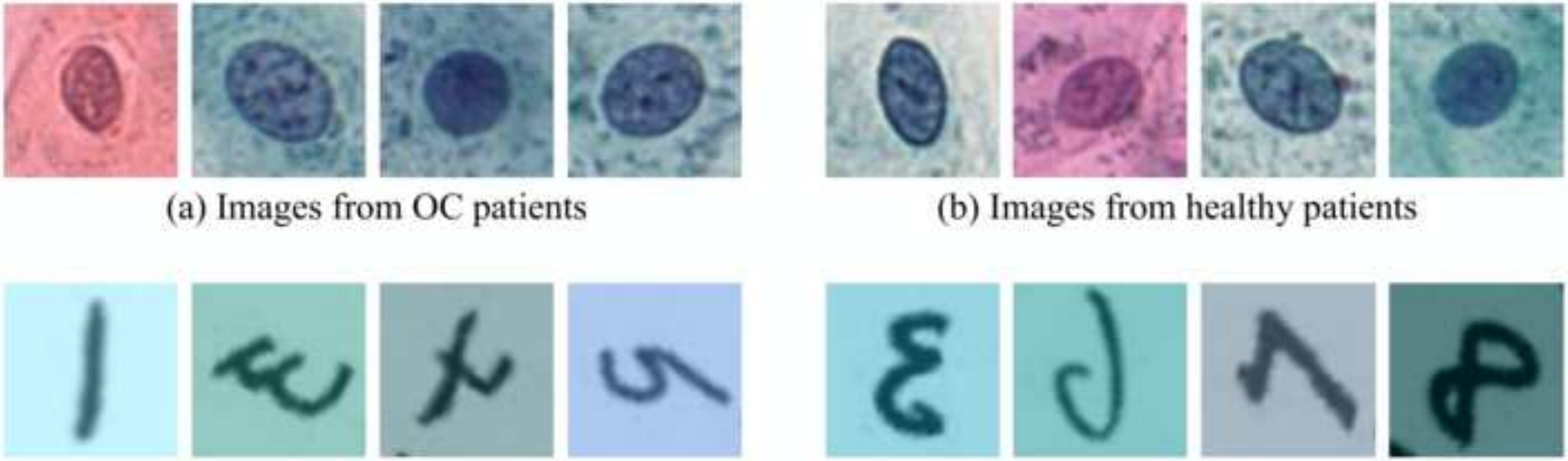
(a) Images from OC patients
(b) Images from healthy patients
(c) Images from a positive bag
(d) Images from a negative bag

Click here to access/download;Figure;Fig2.eps

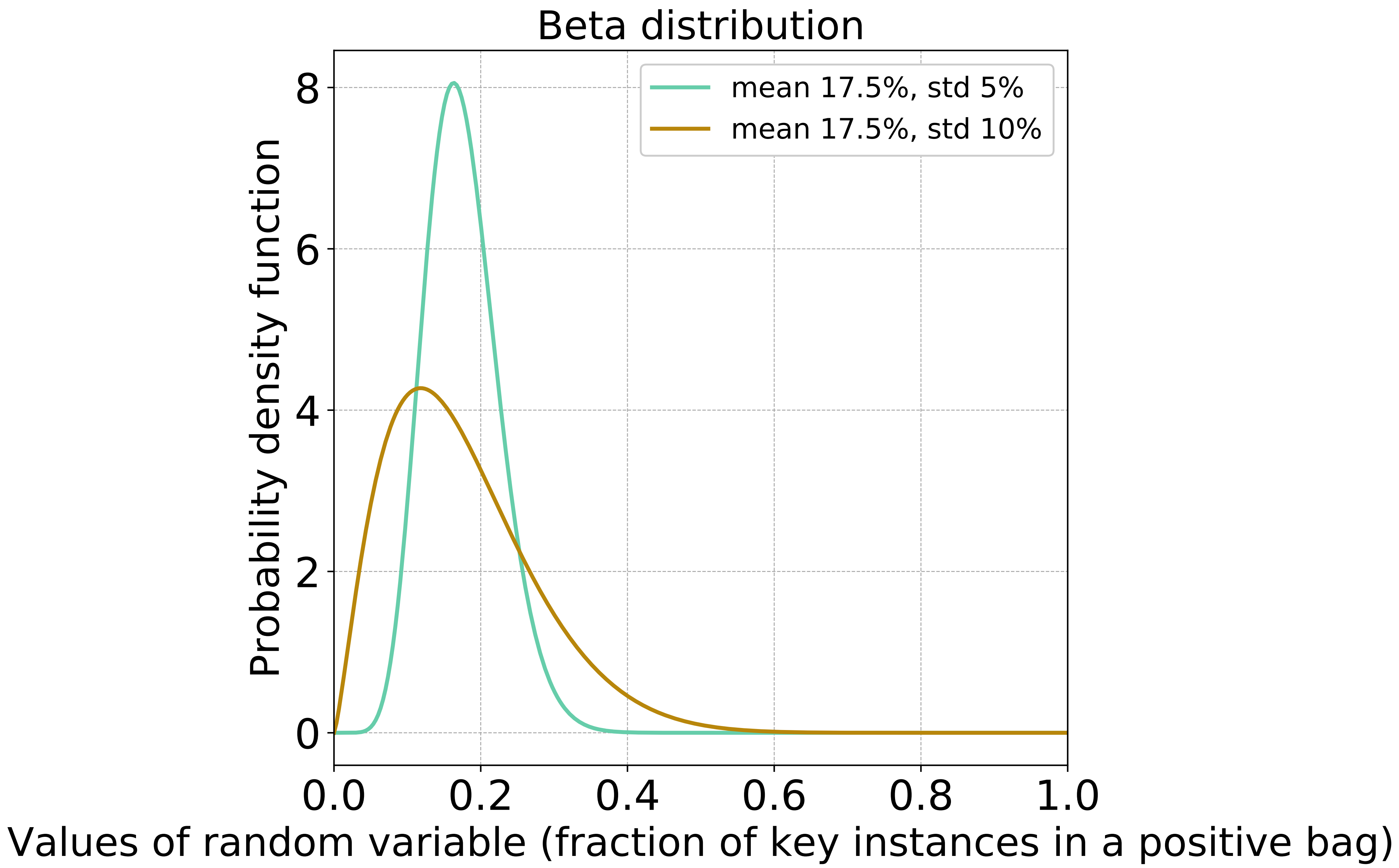

Figure

Click here to access/download;Figure;Fig3.tiff

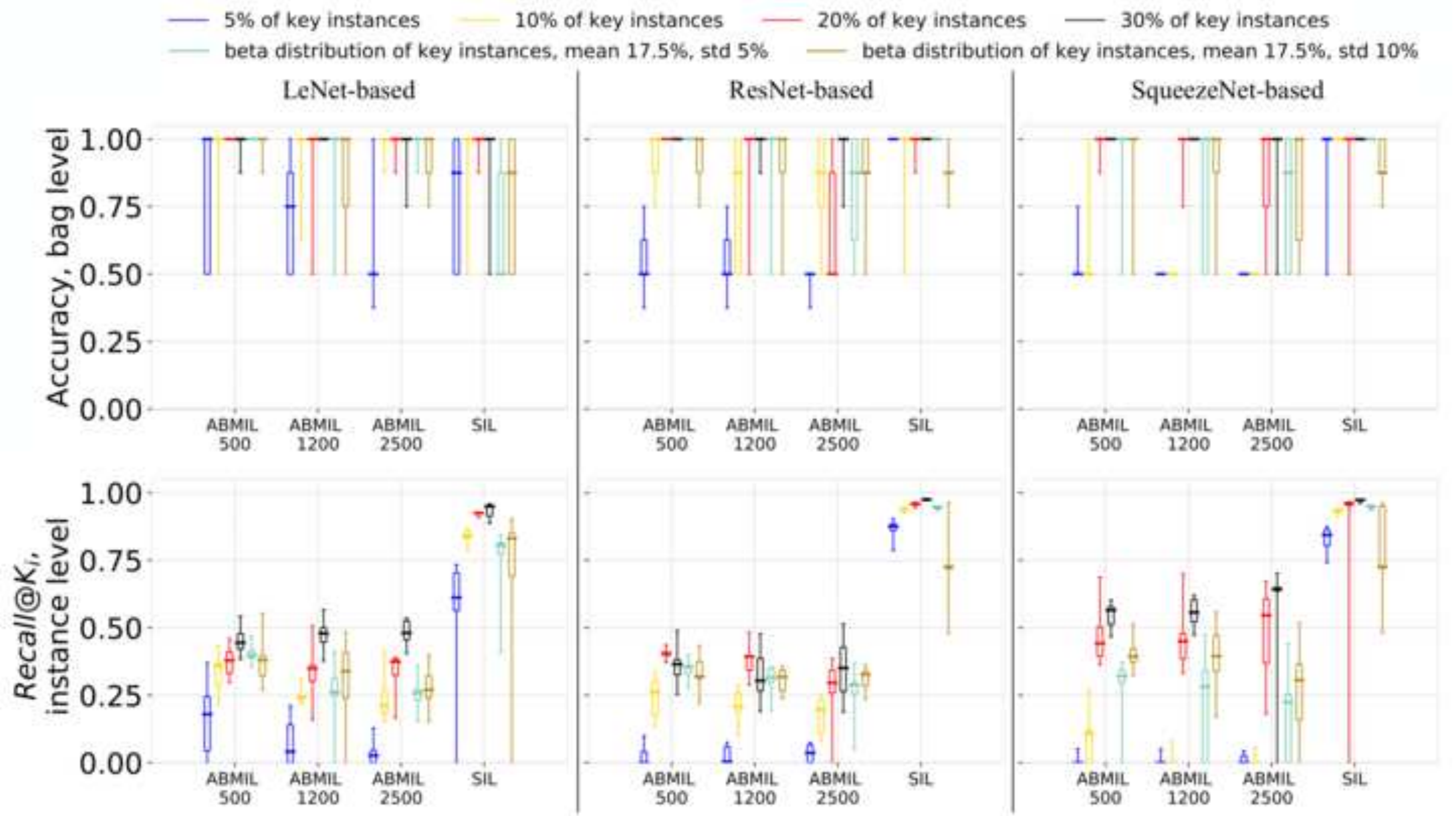

Figure

Click here to access/download;Figure;Fig4.tiff

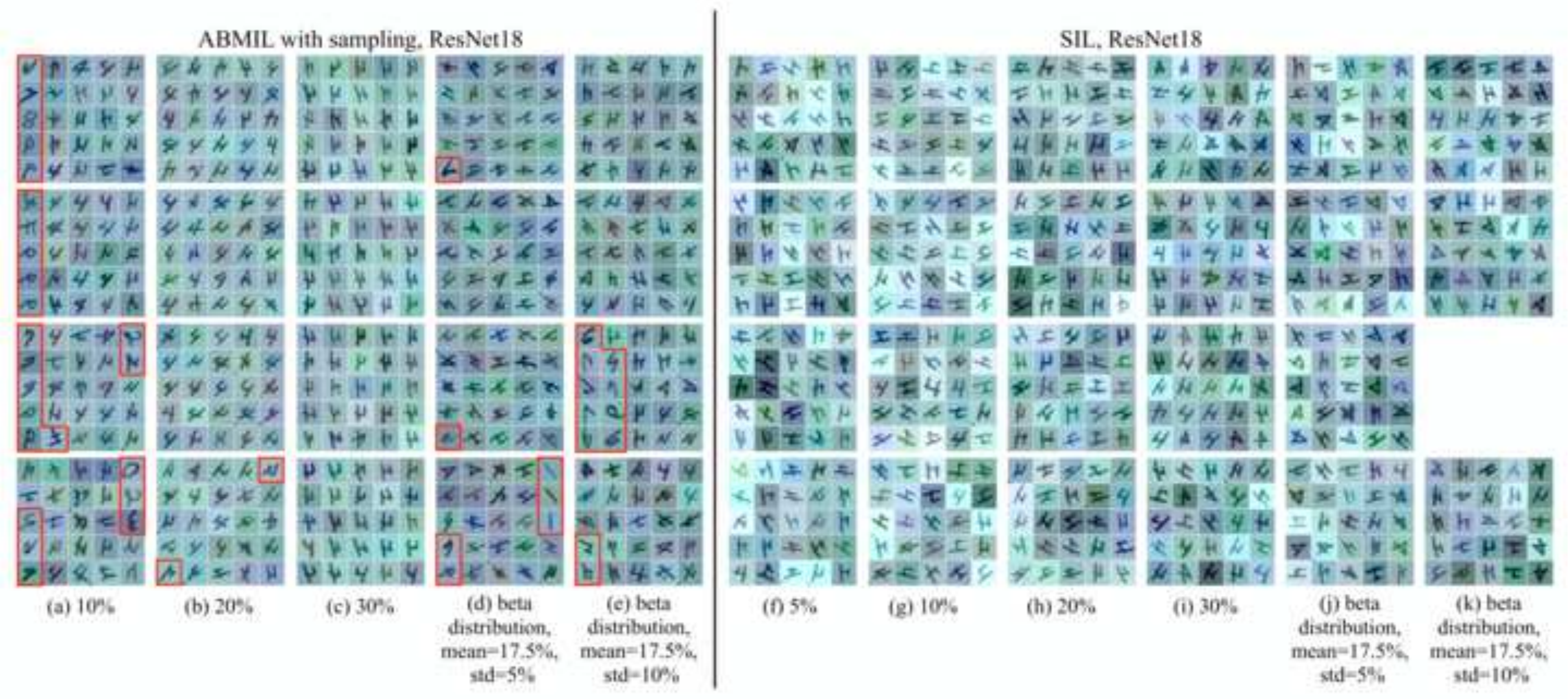

Click here to access/download;Figure;Fig5.tiff

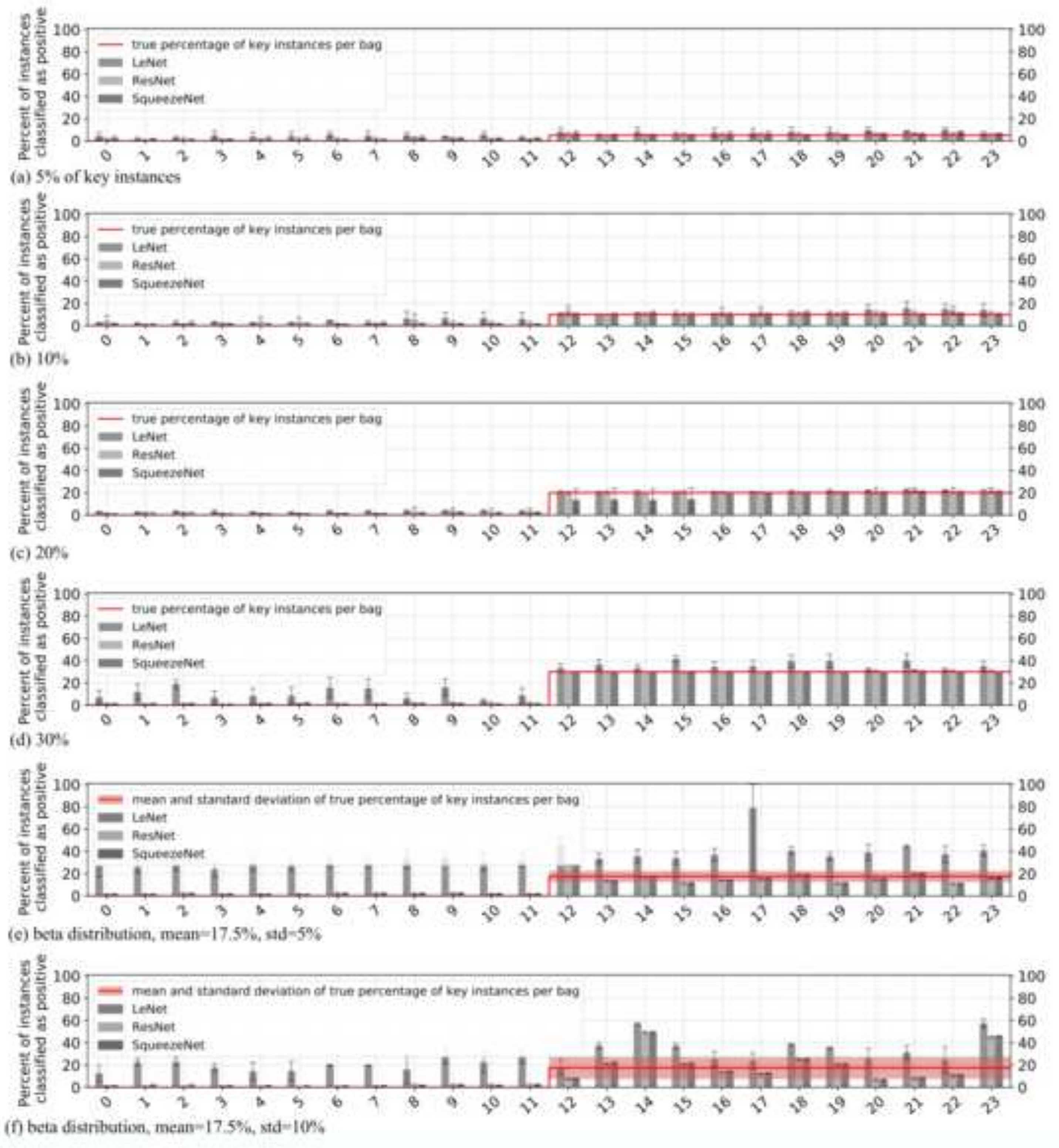

Click here to access/download;Figure;Fig6.eps

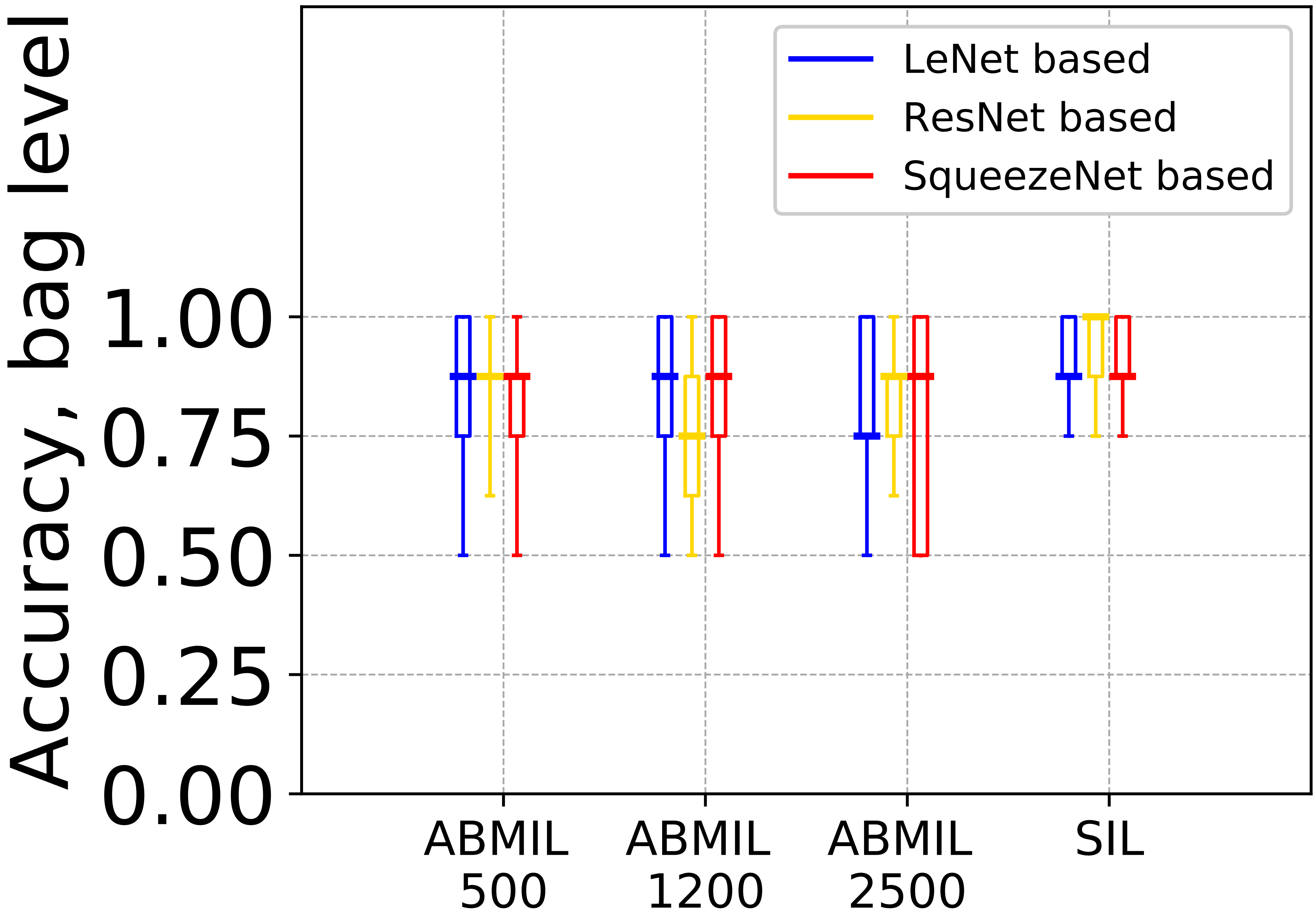

Figure

Click here to access/download;Figure;Fig7.tiff

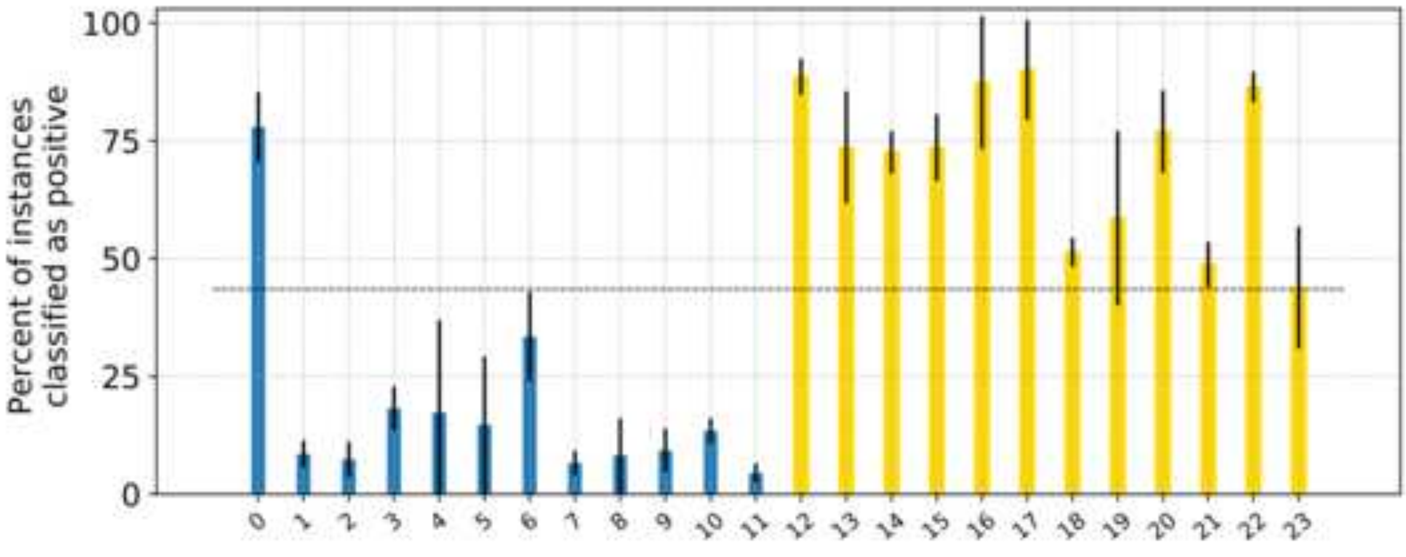

(a) LeNet

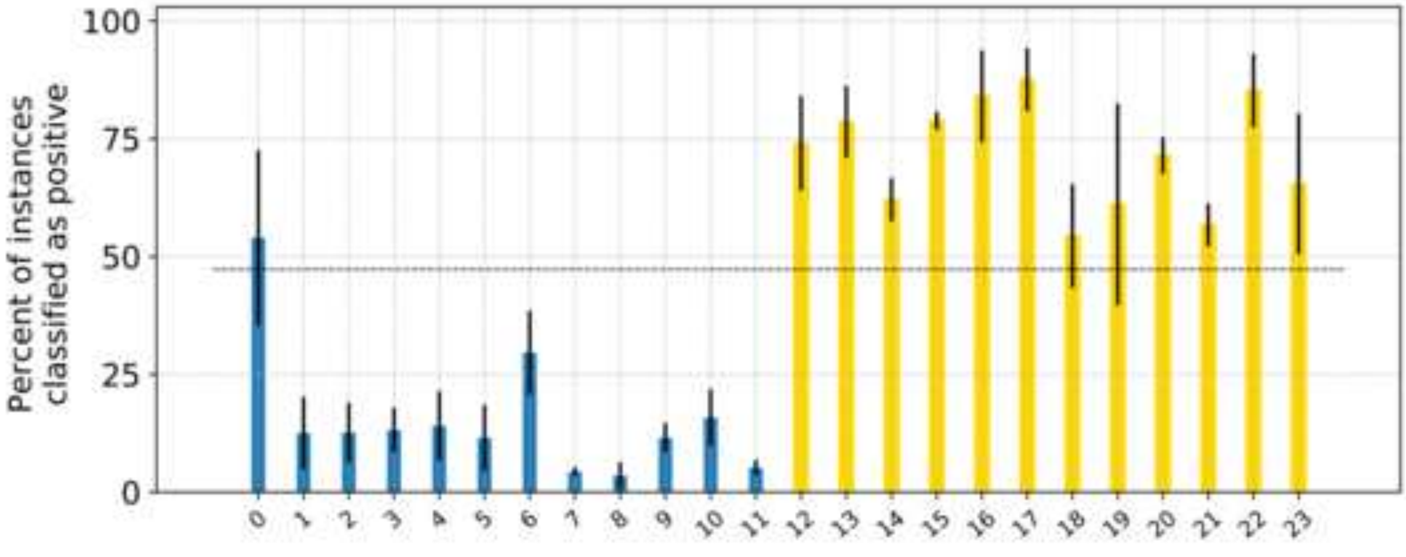

(b) ResNet18

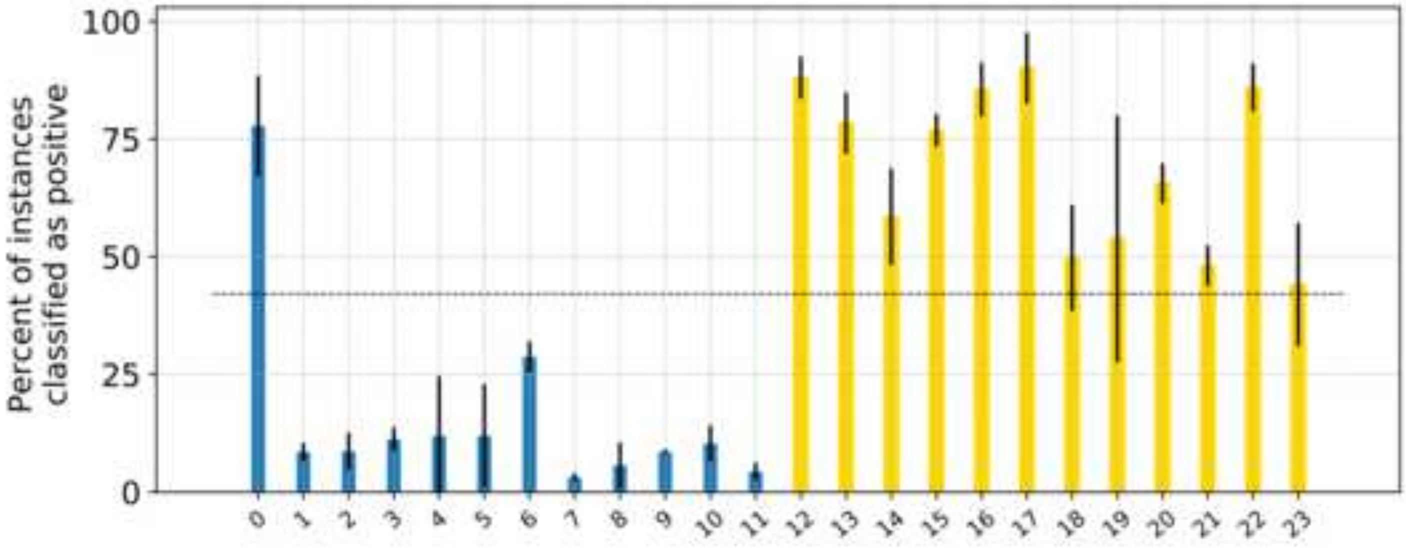

(c) SqueezeNet

Figure
Click here to access/download;Figure;Fig8.tiff

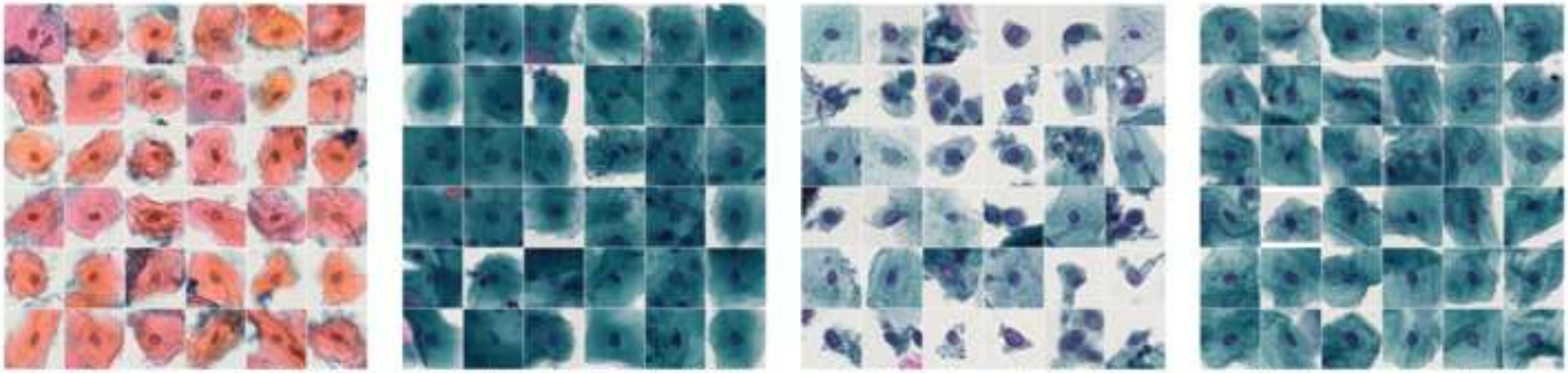

(a) Predicted as positive by ABMIL, all annotated as normal superficial cells by cytotechnologist

(b) Predicted as positive by ABMIL, several cells annotated as ASC-US by cytotechnologist

(c) Predicted as positive by SIL, several cells annotated as suspicious for high grade dysplasia by cytotechnologist

(d) Predicted as negative by SIL, annotated as normal intermediary cells by cytotechnologist

Click here to access/download
Supporting Information
S1_fig.tiff
