# Supplementary material for: Deep multiple instance learning versus conventional deep single instance learning for interpretable oral cancer detection
Source: PLoS One. 2024 Apr 30;19(4):e0302169. doi: 10.1371/journal.pone.0302169 (PMC11060593; doi:10.1371/journal.pone.0302169)
Supplement: Attachment — Submitted filename: Response to Reviewers.pdf [file pone.0302169.s010.pdf]

We thank the reviewers and academic editor for carefully reviewing our manuscript.

Journal Requirements:

1. *Please ensure that your manuscript meets PLOS ONE's style requirements, including those for file naming. The PLOS ONE style templates can be found at*

https://journals.plos.org/plosone/s/file?id=wjVg/PLOSOne_formatting_sample_main_body.pdf and https://journals.plos.org/plosone/s/file?id=ba62/PLOSOne_formatting_sample_title_authors_affiliations.pdf

Authors' response: We (1) removed excessive information of the current address, (2) changed the order of the section Acknowledgements and section Author summary, (3) changed S1 Fig. format in the Supporting information section, (4) changed the format of writing from 'Fig. 1' to 'Fig 1', (5) renamed 'S1_fig.tiff' to 'S1_Fig.tif', (6) changed from 'Dept.' to 'Department', (7) changed section title from 'Author summary' to 'Author Contributions', (8) added initials '(NK)' of the corresponding author after email, (9) use math symbol instead of italics for $\mathrm{Recall@K}_i$.

2. *We note that the grant information you provided in the 'Funding Information' and 'Financial Disclosure' sections do not match.*

When you resubmit, please ensure that you provide the correct grant numbers for the awards you received for your study in the 'Funding Information' section.

Authors' response: We updated the *Funding Information* response to remove sources that are not sources of funding for the work included in this submission. The computational resources provided by NAISS/SNIC are funded separately from the submitted work. The updated *Funding Statement* is: "*This work is supported by: Sweden's Innovation Agency (VINNOVA) https://www.vinnova.se/en/apply-for-funding/funded-projects/, grants 2017-02447 (J.L.), 2021-01420 (J.L.), and 2020-03611 (J.L.), and the Swedish Research Council https://www.vr.se/english/swecris.html#/, grant 2017-04385 (J.L.). The funders had no role in study design, data collection and analysis, decision to publish, or preparation of the manuscript. There was no additional external funding received for this study.*"

3. *Thank you for stating in your Funding Statement:*

"This work is supported by: Sweden's Innovation Agency (VINNOVA) https://www.vinnova.se/en/apply-for-funding/funded-projects/, grants 2017-02447 (J.L.) and 2020-03611 (J.L.), and the Swedish Research Council https://www.vr.se/english/swecris.html#/, grant 2017-04385(J.L.). A part of the computations was enabled by resources provided by the National Academic Infrastructure for Supercomputing in Sweden (NAISS) and the Swedish National Infrastructure for Computing (SNIC) at Chalmers Centre for Computational Science and Engineering (C3SE), partially funded by the Swedish Research Council through grant agreements no. 2022-06725 and no. 2018-05973. The funders had no role in study design, data collection and analysis, decision to publish, or preparation of the manuscript."

*Please provide an amended statement that declares *all* the funding or sources of support (whether external or internal to your organization) received during this study, as detailed online in our guide for authors at http://journals.plos.org/plosone/s/submit-now.*

Please also include the statement "There was no additional external funding received for this study." in your updated Funding Statement.

Please include your amended Funding Statement within your cover letter. We will change the online submission form on your behalf.

Authors' response: The computational resources provided by NAISS/SNIC are funded separately from the submitted work. The updated *Funding Statement* is: "*This work is supported by: Sweden's Innovation Agency (VINNOVA) https://www.vinnova.se/en/apply-for-funding/funded-projects/, grants 2017-02447, (J.L.), 2021-01420 (J.L.), and 2020-03611 (J.L.), and the Swedish Research Council https://www.vr.se/english/swecris.html#/, grant 2017-04385 (J.L.). The funders had no*

role in study design, data collection and analysis, decision to publish, or preparation of the manuscript. There was no additional external funding received for this study."

4. Thank you for stating the following in the Acknowledgments Section of your manuscript:

"This work is supported by: Sweden's Innovation Agency (VINNOVA), grants 2017-02447 and 2020-03611, and the Swedish Research Council, grant 2017-04385. A part of the computations was enabled by resources provided by the National Academic Infrastructure for Supercomputing in Sweden (NAISS) and the Swedish National Infrastructure for Computing (SNIC) at Chalmers Centre for Computational Science and Engineering (C3SE), partially funded by the Swedish Research Council through grant agreements no. 2022-06725 and no. 2018-05973."

Funding information should not appear in the Acknowledgments section or other areas of your manuscript. We will only publish funding information present in the Funding Statement section of the online submission form.

Please remove any funding-related text from the manuscript and let us know how you would like to update your Funding Statement. Currently, your Funding Statement reads as follows:

"This work is supported by: Sweden's Innovation Agency (VINNOVA) https://www.vinnova.se/en/apply-for-funding/funded-projects/, grants 2017-02447 (J.L.) and 2020-03611 (J.L.), and the Swedish Research Council https://www.vr.se/english/swecris.html#/, grant 2017-04385 (J.L.). A part of the computations was enabled by resources provided by the National Academic Infrastructure for Supercomputing in Sweden (NAISS) and the Swedish National Infrastructure for Computing (SNIC) at Chalmers Centre for Computational Science and Engineering (C3SE), partially funded by the Swedish Research Council through grant agreements no. 2022-06725 and no. 2018-05973. The funders had no role in study design, data collection and analysis, decision to publish, or preparation of the manuscript."

Please include your amended statements within your cover letter; we will change the online submission form on your behalf.

Authors' response: We removed any funding-related text from the manuscript. Our updated Funding Statement is: "*This work is supported by: Sweden's Innovation Agency (VINNOVA) https://www.vinnova.se/en/apply-for-funding/funded-projects/, grants 2017-02447 (J.L.), 2021-01420 (J.L.), and 2020-03611 (J.L.), and the Swedish Research Council https://www.vr.se/english/swecris.html#/, grant 2017-04385 (J.L.). The funders had no role in study design, data collection and analysis, decision to publish, or preparation of the manuscript. There was no additional external funding received for this study.*"

5. We noted in your submission details that a portion of your manuscript may have been presented or published elsewhere:

"Preprint of old version in arxiv. Koriakina N, Sladoje N, Bašić V, Lindblad J. Oral cancer detection and interpretation: Deep multiple instance learning versus conventional deep single instance learning. arXiv preprint arXiv:2202.01783. 2022 Feb 3."

Please clarify whether this [publication] was peer-reviewed and formally published. If this work was previously peer-reviewed and published, in the cover letter please provide the reason that this work does not constitute dual publication and should be included in the current manuscript.

Authors' response: The work [1] is an unreviewed preprint. It has not passed a peer review, and has not been formally published. We have introduced modifications into [1] and performed additional experiments before submitting the manuscript to PLOS ONE.

> [1] "*Koriakina N, Sladoje N, Bašić V, Lindblad J. Oral cancer detection and interpretation: Deep multiple instance learning versus conventional deep single instance learning. arXiv preprint arXiv:2202.01783. 2022 Feb 3.*"

6. In your Data Availability statement, you have not specified where the minimal data set underlying the results described in your manuscript can be found. PLOS defines a study's minimal data set as the underlying data used to reach the conclusions drawn in the manuscript and any additional data required to replicate the reported study findings in their entirety. All PLOS journals require that the minimal data set be made fully available. For more information about our data policy, please see http://journals.plos.org/plosone/s/data-availability.

Upon re-submitting your revised manuscript, please upload your study's minimal underlying data set as either Supporting Information files or to a stable, public repository and include the relevant URLs, DOIs, or accession numbers within your revised cover letter. For a list of acceptable repositories, please see http://journals.plos.org/plosone/s/data-availability#loc-recommended-repositories. Any potentially identifying patient information must be fully anonymized.

Important: If there are ethical or legal restrictions to sharing your data publicly, please explain these restrictions in detail. Please see our guidelines for more information on what we consider unacceptable restrictions to publicly sharing data: http://journals.plos.org/plosone/s/data-availability#loc-unacceptable-data-access-restrictions. Note that it is not acceptable for the authors to be the sole named individuals responsible for ensuring data access.

We will update your Data Availability statement to reflect the information you provide in your cover letter.

Authors' response: In our study, we perform comparative evaluation of approaches on two datasets, synthetic PAP-QMNIST data and real world oral cancer data. The codes for generating synthetic PAP-QMNIST data are publicly available as well as the exact versions used in the submitted manuscript. The synthetic PAP-QMNIST data is essential to our study and the majority of the quantitative results and study findings reported in the article are obtained using PAP-QMNIST data. The oral cancer data are not publically available due to ethical permit restrictions. However, we will provide the values used to build graphs, the values behind the means, standard deviations, and other measures reported, as well as the weights for neural network models trained on oral cancer data.

7. We note that you have stated that you will provide repository information for your data at acceptance. Should your manuscript be accepted for publication, we will hold it until you provide the relevant accession numbers or DOIs necessary to access your data. If you wish to make changes to your Data Availability statement, please describe these changes in your cover letter and we will update your Data Availability statement to reflect the information you provide.

Authors' response: We will provide the values used to build graphs, the values behind the means, standard deviations, and other measures, as well as the weights for neural network models trained on oral cancer data and PAP-QMNIST data in Zenodo repository upon acceptance. Our preliminary updated *Data Availability statement: "*PAP-QMNIST data are available from the Zenodo repository, 10.5281/zenodo.7020311. The oral cancer data cannot be shared publicly because of the ethical permit restrictions. The values used to build graphs, the values behind the means, standard deviations, and other measures reported, as well as the weights for neural network models trained on PAP-QMNIST and oral cancer data, are provided in repository XXX."

8. Your ethics statement should only appear in the Methods section of your manuscript. If your ethics statement is written in any section besides the Methods, please move it to the Methods section and delete it from any other section. Please ensure that your ethics statement is included in your manuscript, as the ethics statement entered into the online submission form will not be published alongside your manuscript.

Authors' response: We have inserted the ethics statement as a subsection of the Methods section called "Ethical approval".

Reviewer #1:

Comments:

1) In the abstract, you mention "This dataset serves as a proxy...". This needs to be better explained and describe what you aim to achieve and how you use it.

Authors' response: We added a clarification in the abstract and "Data/PAP-QMNIST Dataset" subsection of the manuscript.

2) Have the authors considered self-supervised methods? Usually there are improvements when combined with MIL approaches. Some results even with simple methods would benefit the paper.

Authors' response: SSL methods typically underperform more supervised AI approaches. In the study, we are applying end-to-end approaches because they are believed to learn better image representations as they are trained for the task of interest in

contrast to image representations learned in an un/selfsupervised, not task-specific way. Even if incorporating SSL-based pretraining (thereby adding complexity to the paper), we see no reason to expect that this would change the relative performance of SIL and MIL approaches.

3) line 58 "comprise" should be "include"

Authors' response: The word 'comprise' is now replaced.

4) Phrase in lines 64-68 should be rewritten to convery the meaning in a more clear manner.

Authors' response: We rephrased and changed the order of the sentences in lines 64-68.

5) In lines 82-83 you write: "A main advantage of PAP-QMNIST is that is offers access to reliable GT annotation at the instance (cell) level". In the previous sentence, you write that GT annotation is not feasible. How both are true? Giving a brief overview of the dataset you created and what you aim to achieve would be beneficial here. Moreover, being more "precise" in the language would avoid confusion (it seems that PAP-QMNIST does not have cells at all).

Authors' response: We agree and we added clarification to the text.

6) In the end of Introduction, I believe "splitting" the contributions and findings, as well as adding a small overview of your findings would be beneficial for the reader (to have a first impression on what to expect from the rest of the paper).

Authors' response: We separated the contributions and findings.

7) Is there a reason for using F1 score instead of metrics like FAR, FRR? These metrics are interpreted in a manner that is easier to associate a cost for each action. An input of a medical expert, e.g. we are interested in very low number of False Negatives, would also add value to your findings.

Authors' response: The SIL model with the highest F1 score in the window with the best moving average F1 score is chosen for evaluation at inference. F1 score is calculated based on weak labels, i.e., instances are given labels of a bag they belong to. F1 score is chosen as a typical measure in case of class imbalance; in our case, imbalance is in the number of instances with positive weak label and negative weak label. The F1 score, being a balanced combination of precision and recall, is used when there is a need for a single scalar to optimize. Since the instance GT is not available for training, but only weak labels, the use of a more tailored metric is not justified and could emphasize, for example, negative instances from positive bags more than negative instances from negative bags. Please note that we use different metrics for evaluation, such as accuracy at the bag level and Precision@K_i at the instance level. We noticed that the name Recall@K_i for the instance metric was misleading and changed it to fit the metric's purpose, which is to quantify the number of real key instances among the top K instances of the bag *i* (assuming that instances with top K weights/scores are predicted as positive). We insert a clarification for the choice of accuracy at the bag level and Precision@K_i at the instance level in the subsection "Experimental setup/Quantitative evaluation" of the manuscript.

8) In section "Considerd MIL method" some description/refresher would help the flow of the paper. For example, what is the modified version described in [13] (again a short description and the reader can refer to [13] for more details)? But throught the paper there isn't any model description.

Authors' response: We made a clarification about the version of ABMIL we use. We included general diagrams of SIL and MIL models in the introduction and added models' details in the Supporting information section.

9) In line 177 you mention 24 patients, and in lines 185-186 you give some extra information. It would help the reader if you transferred the info in 185-186 in line 177.

Authors' response: We agree and move the sentence in lines 185-186 next to the sentence in line 177.

10) In line 194 you write: "Reliable cell-level annotations ... are scarce". In line 199 you write: "while having reliable GT ... at the instance (cell) level. Same as comment 5). This creates confusion.

Authors' response: We added clarification in the paragraph.

11) In line 204 you cite some other datasets. Since your main contribution is the creation of PAP-QMNIST, it would enhance your case if you would show comparative performance of a classification method(s)based on your dataset and those you cited.

Authors' response: MNIST-bags, CIFAR10-bags, QMNIST-bags, and Imagenette-bags datasets are not suitable for such experiments as in our study, as we explain in subsection "Data/PAP-QMNIST Dataset". PAP-QMNIST dataset takes into account the creation of bags with a large number of instances, varying number of instances, and varying percentage of key instances in positive bags as in real world OC data, unlike the above mentioned MIL datasets. It remains unclear to us how the relative performance of PAP-QMNIST and other MIL datasets with instance GT would be useful.

12) In line 215 you write "transformations" expected in OC. It would the reader if you described those, or if you cited a paper that does.

Authors' response: We include more detailed information about transformations that we apply to QMNIST images while creating PAP-QMNIST in the Supporting information section. The transformations are: resizing to image size of 80x80 pixels (probability=1), rotations by multiples of 90 degrees (probability=1), added Gaussian noise (variance range for noise (2,5), probability=0.1) to simulate digital processing inherent error, horizontal flips (probability=1), blur (blur limit=5, probability=0.15) to simulate out-of-focus images, random change of brightness and contrast by a small factor (factor range (-0.2, 0.2), probability=0.8) to simulate the change in brightness and contrast for different parts of a WSI, motion blur (blur limit range (3,3), probability=0.05) to simulate external vibrations that can affect the imaging by a microscope, fog (lower coefficient=0.3, upper coefficient=0.4, alpha coefficient=0.08, probability=0.05) to simulate fog that could appear due to a microscope lens.

13) Have you tried any AI colorization scheme? There are some free tools for this as well. Would be interesting to see if they have any effect compared to your way of colorization the QMNIST images.

Authors' response: In an AI-based colorization scheme, artificial neural networks are first trained on available data (e.g., ImageNet) and then are used to colorize grayscale images. We could use such a colorization scheme for QMNIST data, however, we intended to create PAP-QMNIST as a well-controlled synthetic dataset, and AI-based colorization would not be easily interpretable in contrast to choosing one color for an image. Therefore, incorporating AI-based colorization does not match our objective.

14) In lines 221-222 you write: "...give colorization visually far from colors of OC data". How do you check this? Please include it in paper.

Authors' response: The cells in OC dataset have predominantly blue/turquoise background, therefore, we sample color values from the distributions for each color channel, avoiding values which, in combination with the other two channels, give colorization visually far from blue/turquoise color. We added clarification in the manuscript.

15) In line 225 you write: "if color appears as an important feature... over-fitted the data". Couldn't be that color is an important feature in general and has predictive power? Have you compared the performance with greyscale images? The way you present it is that if color ends up being important then there is over-fitting, which makes one wonder why bother with it then?

Authors' response: We agree that the text could be confusing for the readers, and we have therefore reformulated it to clarify the message we would like to deliver. Color is by explicit and intentional design, not a relevant feature for PAP-QMNIST. However, approaches need to be able to handle colors (regardless of whether they are relevant for classification or not) because color, in general, can carry information about OC data.

The text is reformulated in the manuscript.

16) You need to explain your claim in 236-239. The impact of the ratio could differ between different classification schemes.

Authors' response: We agree with the reviewer that the impact may differ due to the different classification tasks. We have rephrased the statement "This corresponds to evaluation of the impact of the ratio" to "We expect this to be related to the impact of the ratio".

17) Based on your claim in lines 254-258 wouldn't the digit 6 or 9 make a better candidate than 4?

Authors' response: Digits 6 and 9 can be considered as the same class under transformations such as rotation by a random angle and horizontal and vertical flips. Choosing either digit 6 or 9 to represent key instances would result in negative bags to include a large number of instances with the same properties as key instances, which could mislead AI approaches and interpretation by humans. Choosing images of digit 4 to represent key instances would not bring such confusion as the number of images of digits highly similar (close to indistinguishable) to images of digit 4, e.g., some images of digits 7 or 2 depending on the writing style, is much lower than the number of images of either 6 or 9 looking similar to images of either 9 or 6.

18) In line 262 "positive labels" refer to images with digit 4 (though all the tranformations)?

Authors' response: In the study, we evaluate AI approaches on weakly labeled (per-patient/per-bag) data. Positive labels in line 262 refer to all the images in a positive bag, which are transformed images of all digits, not exclusively digit 4. We added the words "weak bag-level" in front of positive and negative labels for clarification.

19) In line 267 you write:"For each key instance setting". What are the different "settings"?

Authors' response: We changed "for each key instance setting" to "for each chosen percentage of key instances in positive bags" for clarification.

20) In line 21 you mention F1 score again. Please consider including other metrics like TPR, FRR, etc.

Authors' response: We insert the answer to Q.7 here: "The SIL model with the highest F1 score in the window with the best moving average F1 score is chosen for evaluation at inference. F1 score is calculated based on weak labels, i.e., instances are given labels of a bag they belong to. F1 score is chosen as a typical measure in case of class imbalance; in our case, imbalance is in the number of instances with positive weak label and negative weak label. The F1 score, being a balanced combination of precision and recall, is used when there is a need for a single scalar to optimize. Since the instance GT is not available for training, but only weak labels, the use of a more tailored metric is not justified and could emphasize, for example, negative instances from positive bags more than negative instances from negative bags. Please note that we use different metrics for evaluation, such as accuracy at the bag level and Precision@K_i at the instance level. We noticed that the name Recall@K_i for the instance metric was misleading and changed it to fit the metric's purpose, which is to quantify the number of real key instances in top K instances of the bag *i* (assuming that instances with top K weights/scores are predicted as positive). We insert a clarification for the choice of accuracy at the bag level and Precision@K_i at the instance level in the subsection "Experimental setup/Quantitative evaluation" of the manuscript."

21) In line 295 you mention that you trained the model for 20 epochs. At first glance, this seems like a low number. Have you tried training for 50 or 100 epochs to see if the behavior changes?

Authors' response: We train SIL for 150 epochs and ABMIL with sampling for 1500 epochs as stated in the section 'Experimental setup'. We save model weights no earlier than epoch 20 to avoid possible fluctuations at the beginning of training. We rephrased the sentence in lines 295-297 (of initially submitted manuscript) to avoid confusion.

22) In line 315 you mention crossover of precision and recall. Since this happens at the instance level wouldn't your data be skewed towards negative samples making precision unreliable? Why did you choose that?

Authors' response: The choice of Precision@K_i (not Recall@K_i - we noticed that the name for the instance metric was not correct and changed it to fit its purpose) is driven by a possible application of similar DL-based approaches in clinical routine. The shortcoming of DL-based weakly supervised approaches is that they do not have a certain threshold of defining whether the instance is detected as positive or not, but rather a continuous range of values for weights/scores. In the absence of per-cell annotations, it would be infeasible for a cytotechnologist to examine all the instances detected as positive at many different thresholds. Instead, a limited number of instances with the top weights/scores would assist a cytotechnologist in decision making, provided that the top instances are diagnostically relevant for oral cancer. Therefore, in our study, we quantify the ability of DL-based approaches to detect the real key instances within top K_i instances in a bag i. We insert a clarification for the choice of measure in the manuscript as well.

23) In line 321, is sampling with replacement or without?

Authors' response: Sampling with replacement is applied during training and inference time to introduce diverse combinations of instances from original bags. We added a clarification in the section "Considered multiple instance learning -- MIL -- method".

24) In lines 330-334, it is not clear why you impose extra conditions. An instance in bag i is considered to be correctly detected as key instance when i and ii and iii hold true. But ii writes that the true label is positive. Isn't that information alone sufficient??

Authors' response: Key instances are only present within positive bags, therefore, the predicted bag label should be positive, otherwise, no key instances are detected by a method. The condition (ii) states that the true instance label should be positive, which means that the real instance label is positive, i.e. the instance is a key instance. Therefore, we need a criterion which would indicate whether the predicted instance label is positive. For that, we have condition (iii), where the predicted instance label is considered positive when the value of attention weight for that instance falls among top K_i weights of the bag i.

For clarity, we decided to change the word 'true' in condition (ii) by the word 'real'.

25) In line 371 put the phrase "for each patient in the test set" at the beginning of the sentence.

Authors' response: We place the phrase "for each patient in the test set" at the beginning of the sentence.

26) In line 381, you write you do not compare human performance .... Why not? Wouldn't that be useful? Such a method could be used as a substitute in places with lack of experts.

Authors' response: Our opinion is that nowadays, AI approaches for OC detection should be considered as a help to experts, not their substitution. It is a long path of AI approaches verification and clinical validation before we reach a fully interpretable and reliable AI system with a performance exceeding human experts that could be considered as a substitute in places with a lack of experts. The inclusion of human expert performance versus the performance of AI approaches would mislead the readers to assume that considered AI approaches are ready for application in clinical routine as a substitute for human experts. We added a new section, "Limitations and future directions" in the manuscript to highlight the challenges of clinical validation of such approaches.

The comparison at the instance level is not possible since we do not have any independent source of true instance-level labels. Even if relying on a group of cythotechnologists, their judgment would still be human-biased.

27) The results section should be combined with the Discussion section to highlight its findings. Currently, it seems that it "aggregates" the captions of tables and figures without offering any insights on what they mean and what value they add to the story of the paper.

Authors' response: We agree and combine Results and Discussion sections to facilitate understanding of the results and avoid repetition in the text.

() This paper would benefit if the authors made a more "concetrated" effort to highlight their contribution, which is the dataset they created and how it can be used to train systems able to detect OC. If they reduced/reorganized figures and tables, it would enable focusing on the dataset and networks they used, which in turn would highlight their findings.

Authors' response: We merged Results and Discussion sections to facilitate understanding of the results and avoid repetition in the text. We also provided more insights into what the results mean.

Reviewer #2:

In my opinion you have to refer in the paper to the updated literature about how the technique can be usefull for oral cancer detection as well for ohter cancer endometrial cancer and in general for all malignanices .Also in infertility as has been demonstrated how machine learning can be helpfull. I suggest you to read and cite these articles:

Circulating miRNAs as a Tool for Early Diagnosis of Endometrial Cancer-Implications for the Fertility-Sparing Process: Clinical, Biological, and Legal Aspects

Fertility-Sparing Strategies for Early-Stage Endometrial Cancer: Stepping towards Precision Medicine Based on the Molecular Fingerprint

The Future Is Coming: Artificial Intelligence in the Treatment of Infertility Could Improve Assisted Reproduction Outcomes-The Value of Regulatory Frameworks

Endometrial Cancer in Reproductive Age: Fertility-Sparing Approach and Reproductive Outcomes

Authors' response: We updated the literature to included following more recent related works in the manuscript:

[2.1] Zhou H, Chen H, Yu B, Pang S, Cong X, Cong L. An end-to-end weakly supervised learning framework for cancer subtype classification using histopathological slides. Expert Systems with Applications. 2024 Mar 1;237:121379.

[2.2] Chen RJ, Wang JJ, Williamson DF, Chen TY, Lipkova J, Lu MY, Sahai S, Mahmood F. Algorithmic fairness in artificial intelligence for medicine and healthcare. Nature Biomedical Engineering. 2023 Jun;7(6):719-42.

[2.3] Medenica S, Zivanovic D, Batkoska L, Marinelli S, Basile G, Perino A, Cucinella G, Gullo G, Zaami S. The Future Is Coming: Artificial Intelligence in the Treatment of Infertility Could Improve Assisted Reproduction Outcomes—The Value of Regulatory Frameworks. Diagnostics. 2022 Nov 28;12(12):2979.

We also included a new section "Limitations and future directions" to point at potential benefits of an AI-based oral cancer detection system as well as at remaining challenges of introducing such a system into clinical practice.

Reviewer #3:

This study seeks to explore AI-based methods for oral cancer (OC) detection, aiming to provide a less invasive alternative to histological examination. By comparing conventional single instance learning (SIL) and modern multiple instance learning (MIL) approaches using real OC data and a synthetic dataset (PAP-QMNIST), the study attempts to determine their effectiveness in identifying malignant and dysplastic cells. However, the study's scope is limited to performance evaluation and lacks a comprehensive analysis of the potential challenges and practical implications of implementing AI-driven OC detection in clinical settings. Additionally, while it compares SIL and MIL methods, it does not explore other AI-based approaches or address the need for interpretability and validation in real clinical scenarios, leaving room for further research in these critical areas.

Authors' response: We realize that there is indeed a need to describe potential challenges and practical implications of implementing AI-driven OC detection in clinical settings and created a new section "Limitations and future directions" in the manuscript.

The choice of the considered methods is inserted in the section "Background and related work". In a nutshell, we do not explore other AI-based approaches because we focus on end-to-end approaches suitable for weakly labeled oral cancer cytology data (with only per-patient annotations) and are able to provide cells relevant for diagnostics.

Reviewer #4:

1) Avoid the extensive use of words like "we", "our"

Authors' response: We are aware that writing in passive form is sometimes considered more appropriate, and we try to avoid using "we" or "our" when it is not necessary. However, explicit expressions help readers to distinguish our contributions/results from contributions/results of previously published works. Such explicit writing is frequently seen in modern literature, both in journal articles [4.1.1] and in conference publications [4.1.2].

> *[4.1.1] Zhou H, Chen H, Yu B, Pang S, Cong X, Cong L. An end-to-end weakly supervised learning framework for cancer subtype classification using histopathological slides. Expert Systems with Applications. 2023 Sep 9:121379*

[4.1.2] Kang M, Song H, Park S, Yoo D, Pereira S. Benchmarking Self-Supervised Learning on Diverse Pathology Datasets. InProceedings of the IEEE/CVF Conference on Computer Vision and Pattern Recognition 2023 (pp. 3344-3354).

2) Don't mention the URL of the code in abstract section

Authors' response: The url is removed from the abstract.

3) The introduction section is too long. It is recommended to summarize this section.

Authors' response: We arrived at the decision that a thorough introduction into the topic and the rest of the paper is beneficial for our manuscript.

4) Figures and Tables captions are too long (in most cases)

Authors' response: We reduced the length of the caption of Fig. 4. Considering the general recommendation for scientific writing that captions should be informative enough such that the associated Figure/Table can be understood when observed stand alone, we believe that the text in captions for the rest of the figures are crucial to understand the figures and are not fitting well in the main text.

5) The number of patients (24) is limited for a comparative study. Explain how could you overcome this limitation.

Authors' response: Indeed, this is a limitation of our study – it prevents us from drawing conclusions regarding more data-rich conditions. For the same reason, we also refrain from drawing any stronger conclusions on the overall performance w.r.t. OC classification. Still, the fairly low number of independent subjects is not at all uncommon in similar types of scientific studies, whereby we consider our results regarding the performance properties of SIL and MIL to remain highly relevant for researchers facing similar (data-scarce) conditions.

6) Why have you chosen certain measures of performance and neglected other measures? Justify

Authors' response:

(1) accuracy at the bag ('patient') level for both PAP-QMNIST and OC datasets is chosen as a standard measure for a balanced number of bags ('patients') in positive and negative classes.

(2) The choice of Precision@K_i (not Recall@K_i - we noticed that the name for the instance metric was not correct and changed it to fit its purpose) is driven by a possible application of similar DL-based approaches in clinical routine. The shortcoming of DL-based weakly supervised approaches is that they do not have a certain threshold of defining whether the instance is detected as positive or not, but rather a continuous range of values for weights/scores. In the absence of per-cell annotations, it could be infeasible to show to a cytotechnologist all the instances detected as positive at many different thresholds. Instead, the limited number of instances with the top weights/scores would assist a cytotechnologist in decision making. Therefore, in our study, we quantify the ability of DL-based approaches to detect the real key instances within top K_i instances in a bag i. We insert a clarification for the choice of measure in the manuscript as well.

7) Give a comparison (in tabulated form) with previous studies in the field (before conclusion section)

Authors' response: There are no direct studies we could compare our work with for the following reasons: (i) there are no studies that aimed at WSI-level analysis of oral cancer cytology data; (ii) there are no previously presented synthetic datasets that could be an alternative to PAP-QMNIST dataset; (iii) a thorough bag (per-patient) and instance (per-cell) level evaluation of end-to-end DL approaches applied to weakly labeled data has not been priorly demonstrated. We believe that we listed the research gaps in the sections Introduction and Data (more specifically, point (i) in lines 46-51 in the not revised manuscript, point (ii) in lines 204-212 in the not revised manuscript, and point (iii) after lines 97-100 in the revised manuscript with track changes.

8) Add (3-5) recent references 2022-2023

Authors' response: We included the following recent references in the manuscript:

[4.8.1] Zhou H, Chen H, Yu B, Pang S, Cong X, Cong L. An end-to-end weakly supervised learning framework for cancer subtype classification using histopathological slides. Expert Systems with Applications. 2024 Mar 1;237:121379.

[4.8.2] Chen RJ, Wang JJ, Williamson DF, Chen TY, Lipkova J, Lu MY, Sahai S, Mahmood F. Algorithmic fairness in artificial intelligence for medicine and healthcare. Nature Biomedical Engineering. 2023 Jun;7(6):719-42.

[4.8.3] Medenica S, Zivanovic D, Batkoska L, Marinelli S, Basile G, Perino A, Cucinella G, Gullo G, Zaami S. The Future Is Coming: Artificial Intelligence in the Treatment of Infertility Could Improve Assisted Reproduction Outcomes—The Value of Regulatory Frameworks. Diagnostics. 2022 Nov 28;12(12):2979.

Reviewer #5:

1. Authors have conducted experiments on a very limited and outdated set of deep learning models, which is insufficient in the current scenario.

Authors' response: The reason why we focused on the chosen, relatively small, models is overfitting. We have used the larger model, ResNet50, however, even with tuning the weight decay coefficient and/or learning rate, we observed large overfitting on a trial PAP-QMINST data. The evaluation in Lu et al. [5.1.1] of DenseNet201 vs ResNet50 models trained on the part of our data showed essentially the same performance. Since we observed overfitting of the ResNet50 model on trial PAP-QMNIST data, we expect that the DenseNet201 and similar deep models would demonstrate overfitting as well. We observed that choosing models with simpler architectures prevented overfitting in a better way. Our experience is in agreement with the studies by Teramoto et al. [5.1.2] and Zhou et al. [5.1.3], where authors observed that models with a simple architecture showed better performance on their cytology/histology data than more complex alternatives.

We also observe that not all the deep learning models are suitable for images of size smaller than 80x80 pixels; input images propagating deep models are eventually reduced to the feature map size of less than 1x1 pixels, which does not allow learning. With the size of OC images 80x80 pixels, an example of such models is InceptionResNetV2.

In general, our main interest in the submitted study is to compare the performance and behavior of different approaches, SIL and MIL, but not different architecture types. Therefore, comparing all existing architechture types is not our primary goal.

[5.1.1] Lu J, Sladoje N, Runow Stark C, Darai Ramqvist E, Hirsch JM, Lindblad J. A deep learning based pipeline for efficient oral cancer screening on whole slide images. InInternational Conference on Image Analysis and Recognition 2020 Jun 17 (pp. 249-261). Cham: Springer International Publishing.

[5.1.2] Teramoto A, Kiriyama Y, Tsukamoto T, Sakurai E, Michiba A, Imaizumi K, Saito K, Fujita H. Weakly supervised learning for classification of lung cytological images using attention-based multiple instance learning. Scientific reports. 2021 Oct 13;11(1):20317

[5.1.3] Zhou H, Chen H, Yu B, Pang S, Cong X, Cong L. An end-to-end weakly supervised learning framework for cancer subtype classification using histopathological slides. Expert Systems with Applications. 2023 Sep 9:121379

2. Merely mentioning ResNet is not enough; it should clearly state which version of the ResNet model was used.

Authors' response: We agree with the comment and added the version of ResNet used in tables and figures where it was missing.

3. The manuscript lacks a state-of-the-art comparison with other models, such as DenseNet 201, EfficientNet-B0 to B7, MobileNet v3, etc.

Authors' response: See our reply to question 1 from reviewer #5 above.

4. There are very limited details about the deep learning models in the manuscript. Please provide more details.

Authors' response: We included general diagrams of SIL and MIL models in the introduction and added models' details in the Supporting information section. We also reformulated the section "Considered multiple instance learning – MIL – method" to facilitate understanding that ABMIL differs from the original version by sampling instances from large bags at training time and inference.

5. *Architectural diagrams of the deep learning model are also missing.*

Authors' response: We included general diagrams of SIL and MIL models in the introduction and added models' details in the Supporting information section.

6. *The details of the hyperparameters in the manuscript are not sufficient to support the reproducibility of the results. Just providing the GitHub link is not enough; crucial details should be in the main manuscript.*

Authors' response: Optimization details are placed in the Supporting information section, and the number of epochs and mini-batch size are in the section Experimental evaluation.

7. *The manuscript does not properly state the research gaps that motivated the proposed work.*

Authors' response: The research gaps that motivated the proposed work are: (i) there are no studies that would aim at WSI-level analysis of oral cancer cytology data; (ii) there are no synthetic datasets that could be an alternative to the PAP-QMNIST dataset; (iii) a thorough bag (per-patient) and instance (per-cell) level evaluation of end-to-end DL approaches applied to weakly labeled data has not been priorly demonstrated. We believe that we listed the research gaps in the sections Introduction and Data (more specifically, point (i) in lines 46-51 in the not revised manuscript, point (ii) in lines 204-212 in the not revised manuscript and point (iii) after lines 97-100 in the revised manuscript with track changes.) We have added a clarification for the point (iii) in the text: 'We use PAP-QMNIST dataset to perform a thorough bag (per-patient) and instance (per-cell) level evaluation of end-to-end DL approaches applied to weakly labeled data that has not been priorly demonstrated.'

8. *There are various grammatical and typographical mistakes in the manuscript. Please correct them.*

Authors' response: We found and corrected several grammatical and typographical mistakes in the manuscript, e.g., mismatch of American and British writing styles, missing articles, missing commas.

9. *A well-designed block diagram to illustrate the complete methodology would enhance the understanding of the manuscript.*

Authors' response: We included general diagrams of SIL and MIL models in the introduction section.

10. *The literature review is quite outdated and not very significant. Please include recent papers. Authors are also encouraged to discuss how the proposed work significantly contributes in the context of those recent works.*

Authors' response: We searched for recent papers and found relevant to our study publications, such as:

[5.10.1] Zhou H, Chen H, Yu B, Pang S, Cong X, Cong L. An end-to-end weakly supervised learning framework for cancer subtype classification using histopathological slides. Expert Systems with Applications. 2024 Mar 1;237:121379.

[5.10.2] Chen RJ, Wang JJ, Williamson DF, Chen TY, Lipkova J, Lu MY, Sahai S, Mahmood F. Algorithmic fairness in artificial intelligence for medicine and healthcare. Nature Biomedical Engineering. 2023 Jun;7(6):719-42.

[5.10.3] Medenica S, Zivanovic D, Batkoska L, Marinelli S, Basile G, Perino A, Cucinella G, Gullo G, Zaami S. The Future Is Coming: Artificial Intelligence in the Treatment of Infertility Could Improve Assisted Reproduction Outcomes—The Value of Regulatory Frameworks. Diagnostics. 2022 Nov 28;12(12):2979.

We included clarifications regarding the contributions in the section "Introduction" and "Background and related work".

11. *Authors should state the limitations of the proposed work and provide directions for future research.*

Authors' response: We agreed with the reviewer and added a new section "Limitations and future directions" in the manuscript.
