# Supplementary material for: Deep multiple instance learning versus conventional deep single instance learning for interpretable oral cancer detection
Source: PLoS One. 2024 Apr 30;19(4):e0302169. doi: 10.1371/journal.pone.0302169 (PMC11060593; doi:10.1371/journal.pone.0302169)
Supplement: Attachment — Submitted filename: Response_to_reviewers.pdf [file pone.0302169.s011.pdf]

A rebuttal letter

We thank the reviewers and academic editor for providing suggestions on improving our publication.

Journal requirements:

1. *Please review your reference list to ensure that it is complete and correct. If you have cited papers that have been retracted, please include the rationale for doing so in the manuscript text, or remove these references and replace them with relevant current references. Any changes to the reference list should be mentioned in the rebuttal letter that accompanies your revised manuscript. If you need to cite a retracted article, indicate the article's retracted status in the References list and also include a citation and full reference for the retraction notice.*

Authors' response: We updated one citation from

[1] Zhou H, Chen H, Yu B, Pang S, Cong X, Cong L. An end-to-end weakly supervised learning framework for cancer subtype classification using histopathological slides. Expert Systems with Applications. 2023; p. 121379.

to

[2] Zhou H, Chen H, Yu B, Pang S, Cong X, Cong L. An end-to-end weakly supervised learning framework for cancer subtype classification using histopathological slides. Expert Systems with Applications. 2024;237:121379.

due to the replacement of a pre-print version of the publication by a version with a publisher's standard format.

Reviewer # 2:

1. *Dear authors,*
I read with great interest the manuscript, which falls within the aim of this Journal. In my honest opinion, the topic is interesting enough to attract the readers' attention. Nevertheless, authors should clarify some points and improve the discussion, as suggested below. Authors should consider the following recommendations:
In my opinion you have to improve the paper refering in the text how its really impoerant to refer and compare to the PAPILLOMAVIRUS and oral cancer correlation and how artifical ointelligence in this denatality ERA can be really importnat especially in pts with cervical and other malignanicues as endometrial cancer that need to preserv their fertility by oocite vitrification for future use before fertility sparing surgery treatment.

Tthe artifical intelligence contribution as well how is suggested to these pts to perform a NIPT TEST at beginning of pregnancy as well to follow up the impart of ASSITSTED REPRODUCTIVE TECHNOLOGY ART) in the newborn.

I SUGGEST YOU TO READ AND CITE THESE ARTICLES:

[3] Gullo G, Perino A, Cucinella G. Open vs. closed vitrification system: which one is safer?. European review for medical and pharmacological sciences. 2022 Feb;26(4):1065-7.

[4] Mutlu L, Manavella DD, Gullo G, McNamara B, Santin AD, Patrizio P. Endometrial cancer in reproductive age: fertility-sparing approach and reproductive outcomes. Cancers. 2022 Oct 22;14(21):5187.

[5] Margioula-Siarkou C, Petousis S, Papanikolaou A, Gullo G, Margioula-Siarkou G, Laganà AS, Dinas K, Guyon F. Neoadjuvant chemotherapy in advanced-stage ovarian cancer–state of the art. Menopause Review/Przegląd Menopauzalny. 2022 Dec 30;21(4):272-5.

[6] Gullo G, Cucinella G, Chiantera V, Dellino M, Cascardi E, Török P, Herman T, Garzon S, Uccella S, Laganà AS. Fertility-Sparing Strategies for Early-Stage Endometrial Cancer: Stepping towards Precision Medicine Based on the Molecular Fingerprint. International Journal of Molecular Sciences. 2023 Jan 3;24(1):811.

[7] Gullo G, Scaglione M, Cucinella G, Chiantera V, Perino A, Greco ME, Laganà AS, Marinelli E, Basile G, Zaami S. Neonatal outcomes and long-term follow-up of children born from frozen embryo, a narrative review of latest research findings. Medicina. 2022 Sep 4;58(9):1218.

[8] Piergentili R, Gullo G, Basile G, Gulia C, Porrello A, Cucinella G, Marinelli E, Zaami S. Circulating miRNAs as a Tool for Early Diagnosis of Endometrial Cancer–Implications for the Fertility-sparing Process: Clinical, Biological and Legal Aspects.

[9] Gullo G, Basile G, Cucinella G, Greco ME, Perino A, Chiantera V, Marinelli S. Fresh vs. frozen embryo transfer in assisted reproductive techniques: a single center retrospective cohort study and ethical-legal implications. European Review for Medical & Pharmacological Sciences. 2023 Jul 15;27(14).

[10] Medenica S, Zivanovic D, Batkoska L, Marinelli S, Basile G, Perino A, Cucinella G, Gullo G, Zaami S. The Future Is Coming: Artificial Intelligence in the Treatment of Infertility Could Improve Assisted Reproduction Outcomes—The Value of Regulatory Frameworks. Diagnostics. 2022 Nov 28;12(12):2979.

[11] Gullo G, Scaglione M, Buzzaccarini G, Laganà AS, Basile G, Chiantera V, Cucinella G, Zaami S. Cell-free fetal DNA and non-invasive prenatal diagnosis of chromosomopathies and pediatric monogenic diseases: A critical appraisal and medicolegal remarks. Journal of Personalized Medicine. 2022 Dec 20;13(1):1.

[12] Gullo G, Scaglione M, Cucinella G, Perino A, Chiantera V, D'Anna R, Laganà AS, Buzzaccarini G. Impact of assisted reproduction techniques on the neuro-psycho-motor outcome of newborns: a critical appraisal. Journal of Obstetrics and Gynaecology. 2022 Oct 3;42(7):2583-7.

Authors' response:

We would like to note to the Reviewer #2 and the editor that we have already cited one of the by Reviewer #2 proposed publications,

[10] Medenica S, Zivanovic D, Batkoska L, Marinelli S, Basile G, Perino A, Cucinella G, Gullo G, Zaami S. The Future Is Coming: Artificial Intelligence in the Treatment of Infertility Could Improve Assisted Reproduction Outcomes—The Value of Regulatory Frameworks. Diagnostics. 2022 Nov 28;12(12):2979.,

referring to it in our section 'Limitations and future directions'.

After a careful consideration of the remaining publications in the proposed literature list, we came to the conclusion that these publications are not relevant enough to the topic and goals of our publication; the insertion of information about *Assisted reproductive technology art* could be misleading for potential readers: we prefer to keep it clear that this paper presents a study on weakly-supervised DL methods applied to oral cancer classification. However, we are grateful for the feedback.

Reviewer #3:
1. *Accept*
All comments have been addressed
I would like to thank the authors for their efforts
the conclusion is justified by the methods and the results

Authors' response: We thank Reviewer #3 for the valuable feedback that improved the paper and for the kind words.

Additional updates:
After a careful self-review, we observed that the way in which we computed the threshold for predicting positive versus negative test bags using SIL by averaging validation thresholds over all the folds could be questioned. The reason for that is that test bags in one fold may appear as train or validation bags of another fold. To make the separation of test and training data unquestionable, we decided to predict bags in a different way, by computing thresholds for each fold separately, without any averaging. Namely, the threshold t_f for each fold f, separating positive and negative test bags, is chosen as the middle value between the fifth percentile (calculated using linear interpolation) of the percentage of instances classified as positive (softmax score>0.5) in positive and negative bags of fold f from train and validation sets. The update has a minor effect on the average and standard deviation of bag accuracy and precision at the instance level, however, it strengthens the overall conclusions of the paper. We have updated the figures and text of our paper correspondingly, with all changes clearly indicated. We have also corrected a few remaining typos and minor linguistic issues.
